# FERONIA Modulates Translational Buffering Capacity of Ribosome-Associated Gene Module in Salt-Stressed Tomato Roots

**DOI:** 10.3390/plants15152278

**Published:** 2026-07-25

**Authors:** Junyu Bai, Yanfen Fan, Ruolin Yang, Jingquan Yu

**Affiliations:** 1 College of Life Sciences, Northwest A&F University, Yangling 712100, China; bai.junyu.bio@gmail.com; 2 Department of Horticulture, Zhejiang University, Zijingang Campus, 866 Yuhangtang Road, Hangzhou 310058, China; yanfen36@illinois.edu; 3 Department of Chemical and Biomolecular Engineering, University of Illinois at Urbana-Champaign, Urbana, IL 61801, USA; 4 College of Horticulture, Northwest A&F University, Yangling 712100, China; 5 Key Laboratory of Horticultural Plants Growth and Development, Agricultural Ministry of China, 866 Yuhangtang Road, Hangzhou 310058, China

**Keywords:** tomato roots, salt stress, FERONIA, ribosome profiling, translatome, translational efficiency, translational buffering, upstream open reading frame

## Abstract

Salt stress limits tomato productivity, yet how translational regulation contributes to root salt adaptation remains poorly understood. We integrated RNA-seq and ribosome profiling in wild-type (WT) and FERONIA (FER) mutant (*fer*) tomato roots under control and 150 mM NaCl conditions. In WT roots, the salt response was predominantly transcript-driven, but a 29-gene ribosome-associated module showed reduced RNA abundance alongside increased translational efficiency, indicating selective translational buffering. FER loss-of-function disrupted this balance, constitutively elevating ribosome occupancy of ribosome-associated genes while reducing basal expression of stress- and ion-transport-related genes; under salt treatment, *fer* also showed stronger ion-transport transcriptional responses but weaker translational efficiency responses of this module. WT salt stress further shifted ribosome allocation from the 5′ untranslated region (UTR) toward the coding sequence (CDS), an effect attenuated in *fer*, alongside positive coupling between uORF and CDS translational efficiency. Feature modeling identified sequence and structural predictors of uORF translation, including weaker local RNA folding near the start codon and specific amino acid and stop codon preferences. Together, these results reveal FER-associated changes in ribosome-associated translational buffering during tomato root salt responses.

## 1. Introduction

Salt stress is one of the most serious abiotic constraints on tomato production because cultivated tomato is generally moderately salt-sensitive: excess NaCl suppresses biomass accumulation, fruit set and yield, and reshapes fruit-quality traits [1,2]. Our previous work in tomato has shown that RBOH1-dependent H_2_O_2_ production contributes to salt stress acclimation and cross-tolerance, highlighting ROS signaling as an established component of tomato salt responses [3,4]. Roots are especially important in this context because they are the first organs to encounter osmotic and ionic stress and help determine Na^+^ exclusion, K^+^ retention, hydraulic adjustment and root-to-shoot signaling [5,6]. In tomato roots, salinity reshapes root system architecture and induces transcriptomic and alternative-splicing reprogramming, supporting roots as an informative tissue for salt-response analysis [7,8].

Plant stress studies have historically relied mainly on transcriptome analyses to define stress-responsive genes and pathways, and recent work has expanded toward multi-omics integration across genomic, proteomic, metabolomic, epigenomic and phenomic layers [9,10,11,12]. However, mRNA abundance often does not fully predict protein abundance or translational output, indicating that post-transcriptional control, particularly translational regulation, can substantially shape plant stress responses. Ribosome profiling (Ribo-seq) has therefore become a key approach because it captures ribosome-protected fragments and, when paired with RNA-seq, enables quantification of ribosome occupancy and translational efficiency (TE) [13,14]. However, Ribo-seq studies of abiotic stress responses remain limited, particularly in crop roots, although examples have been reported for rice salinity, potato drought/heat and lychee cold stress [15,16,17,18]. A tomato root translatome has been mapped under non-stress conditions, demonstrating that Ribo-seq can refine annotation and reveal widespread non-canonical translation in this crop [19], but how salt stress remodels translational regulation in tomato roots remains largely unknown.

FERONIA (FER) belongs to the conserved *Catharanthus roseus* receptor-like kinase 1-like (CrRLK1L) family and acts at the cell surface as a multifunctional signaling hub [20]. FER was first linked to growth control and root hair development through ROP/RAC signaling [21], and later shown to mediate RALF-dependent root growth inhibition [22] and immune-complex organization [23]. In tomato roots, the RALF2–FER–MYB63 module connects FER signaling to growth–defense coordination [24], supporting a role for FER in integrating growth, defense and environmental information rather than acting as a pathway-specific factor. Current reviews and integrated-omics analyses place FER within an extensive signaling network that connects cell-wall status, peptide ligands, hormone crosstalk, Ca^2+^ and ROS signaling, and developmental plasticity [20,25]. In salinity responses, FER maintains cell-wall integrity during salt stress through a Ca^2+^-dependent mechanism and promotes salt tolerance by controlling photorespiratory flux [26,27]. FER has also been linked to translation-related pathways: RALF1–FER phosphorylates eIF4E1 to promote mRNA translation and protein synthesis in Arabidopsis root hairs, and RALF1–FER interacts with and activates TOR signaling under low-nutrient conditions [28,29].

Because FER-associated translational regulation may involve translation initiation, 5′ leaders and upstream open reading frames (uORFs) represent a relevant regulatory layer to examine. uORFs are widespread in plant 5′ leaders and can conditionally regulate downstream main-ORF translation by affecting scanning, reinitiation or ribosome stalling [30]. In plants, uORFs do not act solely as passive repressors: the TBF1 leader links translational control to the growth-to-defense transition, and SAC51/SACL1 transcripts are regulated by thermospermine through uORF-dependent control [31,32]. More broadly, conserved peptide uORFs can mediate stress-responsive translational regulation across plant lineages [33]. Their effects depend on start-codon context, uORF arrangement and spacing, leader architecture, and natural sequence variation [34,35]. When combined with ORF-calling approaches such as RiboCode, PRICE and RiboBA [36,37,38], Ribo-seq data enable translated uORFs to be identified in plant transcriptomes [19,39,40].

Given that translational regulation during the salt stress response in tomato roots remains largely unknown, we addressed this from three angles: coordination among RNA abundance, ribosome occupancy, and translational efficiency during the WT salt response; whether FER loss-of-function disrupts this coordination; and whether 5′ leader ribosome allocation, including its response to FER loss-of-function, and uORF-associated translation represent additional regulatory layers in this system. We treated WT and *fer* seedlings under control conditions or 150 mM NaCl treatment and generated matched RNA-seq and ribosome profiling (Ribo-seq) libraries from root tissue. In WT roots, a 29-gene ribosome-associated module showed reduced RNA abundance alongside increased translational efficiency during the salt response, indicating selective translational buffering; FER loss-of-function constitutively elevated this module’s ribosome occupancy and attenuated its salt-induced translational efficiency response, while also altering ion-transport transcriptional responses. Genome-wide, salt stress shifted ribosome allocation from the 5′ untranslated region toward the coding sequence, an effect attenuated in *fer*; uORF presence was associated with lower coding-sequence translational efficiency, while salt-induced changes in uORF and coding-sequence translational efficiency were positively co-regulated rather than competitive.

## 2. Results

### 2.1. Salt Stress Phenotypes and Quality Assessment of RNA-Seq and Ribo-Seq Libraries

**Figure 1:**
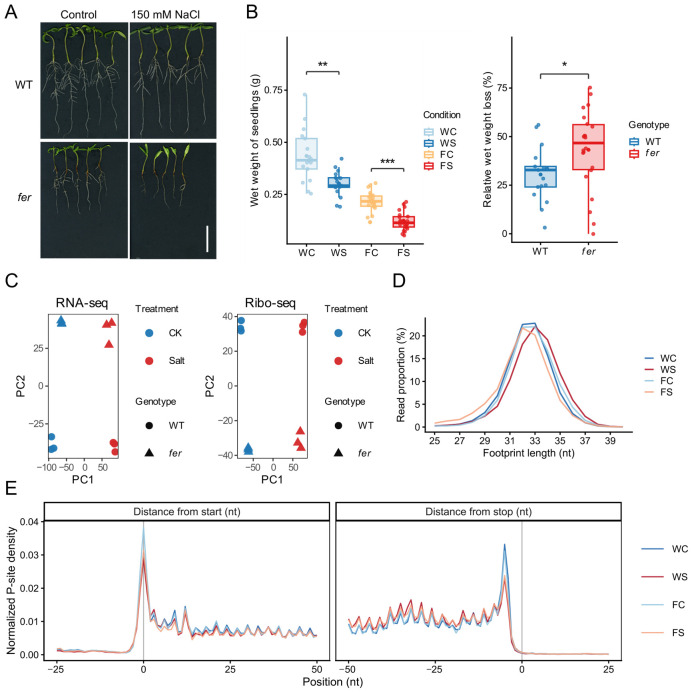
Phenotypes and sequencing quality of WT and *fer* tomato seedlings under control and salt stress conditions. (**A**) WT and *fer* seedlings subjected to control (CK) or 150 mM NaCl (S150) treatment for 5 days. (**B**) Seedling wet weight and relative wet weight change under salt stress across WC, WS, FC and FS conditions. (**C**) Principal component analysis (PCA) of RNA-seq (left) and Ribo-seq (right) gene-level abundance matrices, using the first two principal components (PC1, PC2); point color indicates treatment (CK, blue; Salt, red) and point shape indicates genotype (WT, circle; *fer*, triangle). (**D**) Length distribution of ribosome-protected fragments across the four experimental groups, averaged across biological replicates. (**E**) Metagene profiles of normalized P-site density around annotated start and stop codons, averaged across biological replicates. Condition codes: WC, WT-CK; WS, WT-S150; FC, *fer*-CK; FS, *fer*-S150. Asterisks in panel (**B**) indicate significance levels: * p<0.05, ** p<0.01, *** p<0.001; two-sided Wilcoxon rank-sum tests.

To examine transcriptomic and translatomic responses to FER-associated salt stress regulation, we performed RNA-seq and ribosome profiling using WT and *fer* tomato roots subjected to control (CK) or 150 mM NaCl (S150) treatment for 5 days. The four experimental groups were WT-CK (WC), WT-S150 (WS), *fer*-CK (FC), and *fer*-S150 (FS), each with three biological replicates. To avoid ambiguity, FC refers only to the *fer*-CK condition code in this manuscript; all fold changes are reported as log_2_FC. Under control conditions, *fer* seedlings already showed reduced wet weight compared with WT, indicating a basal growth defect (Figure 1A,B). After 5 days of salt treatment, growth was further inhibited in both genotypes; however, the relative wet-weight reduction was significantly greater in *fer* than in WT (Figure 1B), indicating enhanced salt sensitivity of the mutant.

In total, approximately 361 million, 402 million, 390 million, and 377 million ribosome profiling reads were obtained from WC, WS, FC, and FS samples, respectively. Corresponding RNA-seq libraries yielded approximately 81 million, 67 million, 70 million, and 65 million reads, respectively (Tables S1 and S2). After rRNA removal, 24.79–47.43% of Ribo-seq reads mapped to the tomato reference genome (SL4.0/ITAG4.0). Pairwise Pearson correlation of transcript abundance and ribosome occupancy showed high within-condition reproducibility, with $R^{2}>0.95$ for RNA-seq and $R^{2}>0.93$ for Ribo-seq libraries across biological replicates (Supplementary Figure S1A). Principal component analysis further confirmed that replicates clustered closely within conditions and that samples separated clearly by treatment and genotype, with the treatment effect stronger than the genotype effect at both regulatory levels (Figure 1C).

Ribosome profiling data showed the expected characteristics of high-quality, actively translating libraries. Ribosome-protected fragments (RPFs) were mainly enriched at 31–35 nt, with the dominant peak around 33 nt across all four groups (Figure 1D). Frame 0 was the dominant reading frame in all groups, accounting for approximately 38.9–40.3% of CDS P-sites, consistent with three-nucleotide periodicity (Supplementary Figure S1B). Metagene analysis showed a sharp P-site peak at annotated start codons and enrichment near stop codons, supporting reliable P-site calibration (Figure 1E). Ribo-seq P-sites were predominantly assigned to CDS regions (96.2–96.8% across the four groups), whereas RNA-seq reads were more broadly distributed across 5′UTR, CDS, and 3′UTR regions (Supplementary Figure S1C). Together, these results establish that *fer* shows enhanced salt sensitivity at the phenotypic level and confirm that the RNA-seq and Ribo-seq datasets are of sufficient quality and reproducibility for the transcriptome- and translatome-level analyses that follow.

### 2.2. Regulatory Classification of the WT Salt Response Reveals Translational Buffering of Ribosomal Genes

Salt stress caused extensive reprogramming of both RNA abundance and ribosome occupancy in WT roots. In the WS vs. WC comparison, we identified 1174 upregulated and 1183 down-regulated genes at the RNA-abundance layer, with adjusted p<0.05 and $|\log_{2}\mathrm{FC}|\geq 1$ as cutoffs. At the ribosome-occupancy layer, 1238 genes were upregulated and 1120 genes were down-regulated using the same criteria (Supplementary Figure S2A; Table S3). Among the salt-induced genes, 1016 were shared between the RNA-abundance and ribosome-occupancy layers, representing 72.8% of the union of upregulated genes detected at either layer. Similarly, among the salt-repressed genes, 939 were shared between the two layers, representing 68.8% of the corresponding union of down-regulated genes. However, many genes were regulated at only one layer (Supplementary Figure S2B), indicating layer-specific regulation beyond transcriptional control.

**Figure 2:**
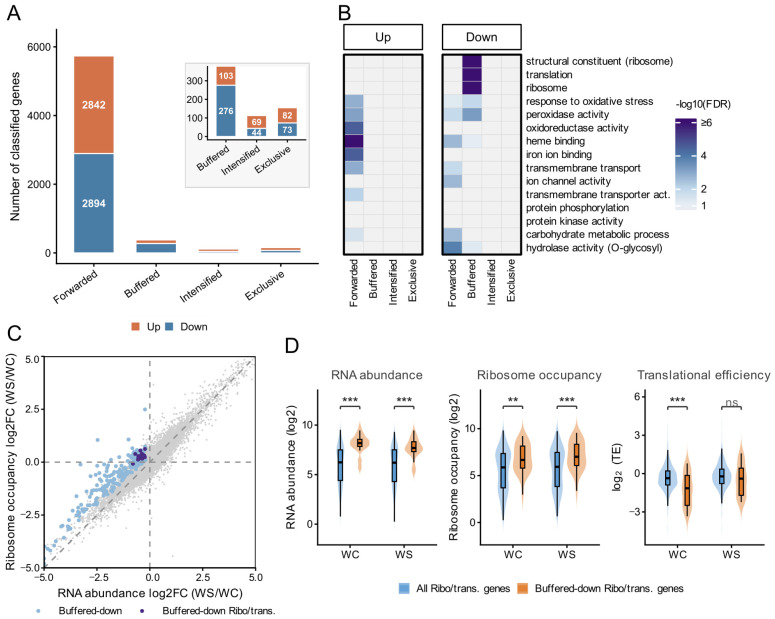
Transcription–translation concordance of salt-responsive genes in WT tomato roots. (**A**) Genes responsive in the WS vs. WC comparison classified into four regulatory classes. Up and down subsets for Forwarded, Buffered and Intensified classes are defined by RNA log_2_FC direction; for the Exclusive class, by ribosome-occupancy log_2_FC direction. (**B**) Gene Ontology enrichment of genes in each regulatory class. Colored cells indicate enriched terms (FDR <0.10); color intensity represents $-\log_{10}\left(\mathrm{FDR}\right)$, capped at 6. (**C**) RNA and ribosome-occupancy changes, highlighting Buffered-down genes with and without ribosome/translation GO annotation. (**D**) RNA abundance, ribosome occupancy, and translational efficiency of ribosome/translation-annotated genes and their Buffered-down subset under WC and WS conditions. Asterisks in panel (**D**) indicate ** p<0.01, *** p<0.001; two-sided Wilcoxon rank-sum tests. ns: no significance.

To characterize how RNA abundance and ribosome occupancy were coordinated, we applied the deltaTE framework [14]. This framework classifies genes into four regulatory categories based on the significance of changes in RNA abundance, ribosome occupancy and translational efficiency, using adjusted p<0.05 but without applying the additional fold-change cutoff used above for differentially expressed gene (DEG) calling (Supplementary Figure S3A): Forwarded, in which ribosome occupancy tracks mRNA changes without a significant translational efficiency change; Buffered, in which translational efficiency changes oppose mRNA changes and attenuate ribosome-occupancy changes; Intensified, in which translational efficiency changes reinforce mRNA changes; and Exclusive, in which significant translational efficiency and ribosome-occupancy changes occur without a significant mRNA change. The Forwarded class was by far the predominant category in both directions, with 2842 upregulated and 2894 down-regulated genes (Figure 2A), indicating that transcriptional reprogramming is the dominant driver of the WT salt response. In contrast, the Buffered (103 up, 276 down), Intensified (69 up, 44 down), and Exclusive (82 up, 73 down) classes represented smaller subsets involving additional translational regulation (inset, Figure 2A; Table S3).

GO enrichment analysis further resolved the functional categories represented by each regulatory class (Figure 2B; Table S4). Forwarded-up genes were enriched for redox- and membrane transport-related functions, whereas Forwarded-down genes were enriched for carbohydrate metabolism, O-glycosyl hydrolase activity, and selected transport-related terms. The clearest signal was observed in the Buffered-down class, in which structural constituents of the ribosome, translation, and the ribosome were among the most significant terms, reaching the display cap of $-\log_{10}\mathrm{FDR}=6$. This enrichment indicated that ribosome- and translation-associated transcripts were preferentially represented among genes whose RNA abundance decreased under salt stress but whose translational efficiency increased, a pattern expected to attenuate the corresponding decrease in ribosome occupancy.

To examine the gene-level behavior underlying this enrichment, we highlighted Buffered-down genes in the genome-wide RNA versus ribosome-occupancy log_2_FC scatter plot and further marked those annotated with ribosome/translation GO terms (Figure 2C; Table S6). Buffered-down genes occupied the region with negative RNA changes accompanied by smaller-magnitude ribosome-occupancy changes, consistent with translational efficiency increases attenuating the effect of transcript repression. The ribosome/translation-annotated subset followed this same buffered pattern, but a larger proportion of these genes showed increased ribosome occupancy in WS relative to WC than did the full Buffered-down class (28 of 29 vs. 130 of 276), despite reduced RNA abundance in WS. Because these enriched Buffered-down genes represented only a subset of all ribosome/translation-annotated genes, we next asked whether they differed from the broader set of ribosome/translation-annotated genes in RNA abundance, ribosome occupancy, and translational efficiency under WC and WS conditions (Figure 2D). The Buffered-down subset showed higher RNA abundance and ribosome occupancy than the broader ribosome/translation gene set under both conditions, indicating that these genes constitute a highly expressed ribosome/translation submodule. Notably, this subset had significantly lower translational efficiency under WC, whereas its translational efficiency became comparable to that of the broader ribosome/translation gene set under WS. Together, these results indicate that these 29 ribosome/translation-annotated Buffered-down genes form a prominent WT salt-response module in which salt-associated RNA reduction is accompanied by increased translational efficiency and, for most genes, increased ribosome occupancy. The net effect of this regulation is therefore enhanced, rather than reduced, ribosome loading despite the accompanying transcript decline.

### 2.3. Loss-of-Function Fer Mutant Shifts Basal Ribosome/Translation Gene Expression and Attenuates Salt-Induced Translational Buffering

Having defined the transcription–translation regulatory landscape of the WT salt response, including the Buffered-down ribosome/translation module, we next asked whether FER loss-of-function had already altered this landscape before salt exposure. We therefore compared WT and *fer* roots under control conditions (FC vs. WC) to identify pre-existing FER-associated differences in RNA abundance, ribosome occupancy, and translational efficiency. In this comparison, *fer* roots differed from WT at both regulatory layers: 384 upregulated and 293 down-regulated genes were detected at the transcript level, and 376 upregulated and 316 down-regulated genes were detected at the ribosome-occupancy level (Supplementary Figure S2C), with 67.4% and 51.1% of induced and repressed genes shared across both layers, respectively (Supplementary Figure S2D). Most classified genes belonged to the Forwarded class (*n* = 2945), with minimal representation in the Buffered (n=10), Intensified (n=3), and Exclusive (n=17) classes (Supplementary Figure S3B), indicating that FER loss-of-function altered basal expression mainly through coordinated RNA and ribosome-occupancy changes rather than widespread translational efficiency remodeling.

**Figure 3:**
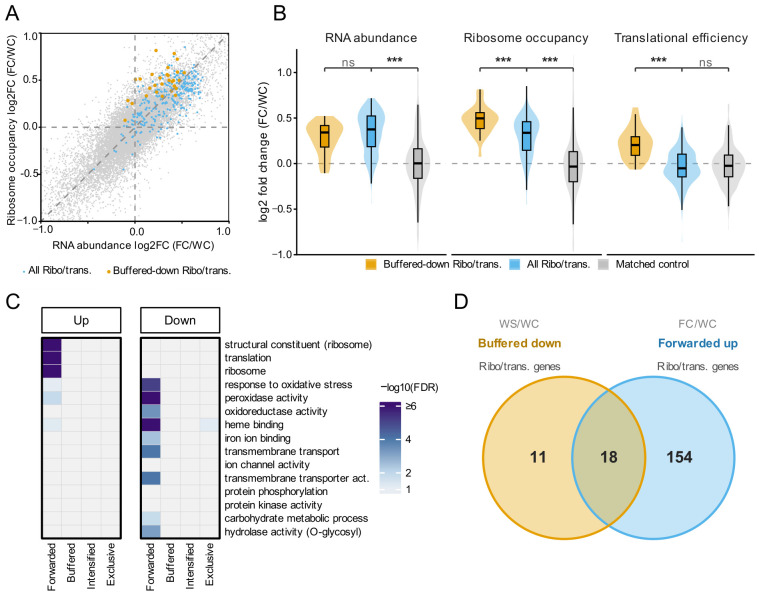
Basal transcriptional and translational differences between WT and *fer* tomato roots. (**A**) RNA and ribosome occupancy changes in the FC/WC comparison, highlighting ribosome/translation GO-annotated genes and the buffered-down subset identified from the WS/WC comparison. (**B**) RNA abundance, ribosome occupancy and translational efficiency fold changes for the buffered-down ribosome/translation subset, all ribosome/translation GO-annotated genes and matched control genes. (**C**) Gene Ontology enrichment of genes in each regulatory class. Colored cells indicate enriched terms (FDR <0.10); color intensity represents $-\log_{10}\left(\mathrm{FDR}\right)$, capped at 6. Up/down subsets for Forwarded, Buffered and Intensified classes are defined by RNA log_2_FC direction; for the Exclusive class, by Ribo log_2_FC direction. (**D**) Overlap between WS/WC buffered-down and FC/WC forwarded-up ribosome/translation genes. Asterisks in (**B**): *** p<0.001; Wilcoxon rank-sum tests for Buffered-down versus all ribosome/translation genes and paired Wilcoxon tests for all ribosome/translation genes versus matched controls. ns: no significance.

GO enrichment analysis of the Forwarded-up class revealed a notable functional signal: structural constituent of ribosome, translation, and ribosome were the top enriched terms ($-\log_{10}\mathrm{FDR}\geq 6$; Figure 3C). This indicated that ribosome/translation-associated genes were co-upregulated at both RNA and ribosome-occupancy levels in *fer* roots at baseline. The Forwarded-down class was enriched for heme binding, peroxidase activity, oxidative-stress response, and ion/transmembrane transport-related functions (Figure 3C), suggesting reduced basal expression of stress- and transport-associated genes in *fer*. This basal reduction may reflect a weakened stress-preparedness state in *fer* roots, potentially contributing to their enhanced sensitivity to subsequent salt exposure.

To determine whether this basal shift overlapped with the 29-gene WT salt-buffered ribosome/translation module, we compared the WS vs. WC Buffered-down and FC vs. WC Forwarded-up ribosome/translation subsets (Figure 3D). Of the 29 Buffered-down ribosome/translation genes, 18 (62%) were also classified as Forwarded-up in FC vs. WC, whereas 154 additional FC vs. WC Forwarded-up ribosome/translation genes fell outside the Buffered-down set. Thus, the FER-associated basal shift overlaps substantially with, but is not limited to, the WT salt-responsive ribosome/translation module.

To resolve the gene-level behavior underlying this overlap, we examined how RNA abundance and ribosome occupancy changed in the FC vs. WC comparison for all ribosome/translation GO-annotated genes and for the 29-gene ribosome/translation subset. In the FC vs. WC RNA versus ribosome-occupancy scatter plot, 246 of the 284 ribosome/translation GO-annotated genes (86.6%) were located in the upper-right quadrant, consistent with the enrichment of ribosome/translation terms in the FC vs. WC Forwarded-up class (Figure 3A). The Buffered-down subset showed a more distinct pattern: 26 of the 29 genes (89.7%) lay above the diagonal, indicating that ribosome-occupancy increases exceeded RNA increases and implying a positive translational efficiency shift in this subset.

To quantify these layer-specific patterns in FC vs. WC, we compared log_2_FC values for RNA abundance, ribosome occupancy, and translational efficiency among the WS vs. WC Buffered-down ribosome/translation subset, all ribosome/translation GO-annotated genes, and ribosome-occupancy-matched control genes (Figure 3B). As a broad class, ribosome/translation GO-annotated genes showed significantly higher RNA-abundance and ribosome-occupancy log_2_FC values than matched controls (p<0.001), but no significant translational efficiency difference, consistent with coordinated RNA and ribosome-occupancy upregulation without a broad translational efficiency shift. In contrast, the 29-gene Buffered-down subset showed significantly higher ribosome-occupancy and TE log_2_FC values than the broader ribosome/translation gene set (p<0.001), whereas RNA-abundance log_2_FC values were comparable between the two groups.

Beyond these basal differences, we next asked whether FER loss-of-function also affects how these 29 ribosome/translation genes buffer salt-induced RNA changes at the ribosome-occupancy level. To quantify buffering directly, we calculated a per-gene buffering index, 1−(ΔRibo/ΔRNA), for the 29 module genes in each genotype, where Δ denotes the salt-induced change in log_2_-transformed RNA abundance or ribosome occupancy within that genotype (WS minus WC in WT; FS minus FC in *fer*). This index measures the extent to which RNA changes are offset at the ribosome-occupancy layer: 0 indicates no buffering, 1 indicates complete buffering, and values >1 indicate overcompensation, in which ribosome occupancy increases despite reduced RNA abundance. 28 of the 29 module genes in WT had an index above 1 (mean =1.68), indicating overcompensation rather than merely compensating for RNA decreases in most module genes. In *fer*, the mean index fell to 0.72, with every gene showing a lower index than in WT (paired Wilcoxon signed-rank test, $p<1\times 10^{-4}$; Supplementary Figure S8C). These results indicate that these 29 module genes in WT robustly overcompensate for salt-induced RNA loss at the ribosome-occupancy level, whereas this compensation is substantially reduced in *fer*.

Because the module already showed elevated baseline translational efficiency in *fer* (Figure 3B), its weaker salt-induced translational efficiency increase could reflect a baseline-dependent effect rather than a FER-dependent contribution to buffering. We tested this possibility using a split-half design in which baseline translational efficiency and salt-induced ΔTE were estimated from non-overlapping replicates (Supplementary Figure S8A,B). After correcting for each genotype’s genome-wide baseline–response trend, the 29-gene module still showed greater-than-expected ΔTE in both genotypes (permutation $p<1\times 10^{-4}$ in WT and p=0.0087 in *fer*), with a significantly larger excess response in WT than in *fer* (module residual +0.69 vs. +0.21; Mann–Whitney test, p<0.0001). Because baseline and response were estimated from non-overlapping replicates, and because the excess response was measured against each genotype’s own background trend, this difference is unlikely to be explained by shared measurement noise or by the baseline shift alone.

A complementary genotype contrast supported this interpretation. The module’s genotype translational efficiency difference, measured as TE log_2_FC (*fer*/WT; positive values indicate higher translational efficiency in *fer* than in WT), was calculated separately under control and salt conditions. This contrast reversed sign from +0.22 under control to −0.29 under salt (paired Wilcoxon signed-rank test, $p<1\times 10^{-4}$; sign reversed in 25 of 29 genes; Supplementary Figure S8D). If FER loss-of-function only elevated basal translational efficiency independently of salt-induced buffering, the elevated *fer*/WT translational efficiency difference would be expected to persist under salt, and translational efficiency of the module genes in FS should remain comparable to or higher than that in WS. Instead, *fer* roots switched from higher translational efficiency of the module genes under control to lower translational efficiency under salt, indicating that FER loss-of-function attenuates the module’s salt-induced buffering beyond what can be explained by the elevated baseline alone.

### 2.4. Genotype-by-Salt Interaction Reveals FER-Associated Ion Transport and Ribosome-Related Translation

Having established this targeted module-level effect, we next asked whether FER-associated salt responsiveness could also be detected at a broader functional-response level. The pairwise comparisons above characterized the WT salt response and the *fer* basal transcription–translation state, but they cannot by themselves isolate functional categories with genotype-dependent salt responses. We therefore first used the FS vs. FC and FS vs. WS pairwise comparisons as descriptive context and then applied genotype-by-salt interaction analysis to identify FER-associated functional responses across the RNA-abundance, ribosome-occupancy, and translational efficiency layers.

Salt treatment of *fer* roots (FS vs. FC) elicited widespread changes at both regulatory layers: 894 up- and 613 down-regulated genes were identified at the RNA-abundance level, and 977 up- and 521 down-regulated genes were identified at the ribosome-occupancy level (Supplementary Figure S2E,F). Most classified genes belonged to the Forwarded class (*n* = 3646), with smaller Buffered (n=63), Intensified (n=46), and Exclusive (n=60) classes (Supplementary Figure S3C); each class contained fewer genes than its counterpart in the WS vs. WC comparison. Unlike the WT salt response, however, the FS vs. FC comparison did not show ribosome/translation enrichment in the Buffered-down class; the major enriched terms were instead associated with membrane, redox, defense, and cell-wall functions (Supplementary Figure S4A; Table S4). The direct genotype comparison under salt conditions (FS vs. WS) addressed a distinct question: how salt-treated *fer* roots differed from salt-treated WT roots. In this comparison, 360 up- and 279 down-regulated genes were identified at the RNA-abundance level, and 325 up- and 257 down-regulated genes were identified at the ribosome-occupancy level (Supplementary Figure S2G,H). This comparison was also dominated by the Forwarded class (*n* = 2385), with smaller Buffered (n=27), Intensified (n=6), and Exclusive (n=16) classes (Supplementary Figure S3D); GO enrichment highlighted ribosome/translation terms in Forwarded-up genes, redox-related functions, heme binding, and iron binding in Forwarded-down genes, and ion-transport terms in Buffered-up genes (Supplementary Figure S4B; Table S4). Together, these pairwise comparisons set the stage for the genotype-by-salt interaction analysis presented next.

**Figure 4:**
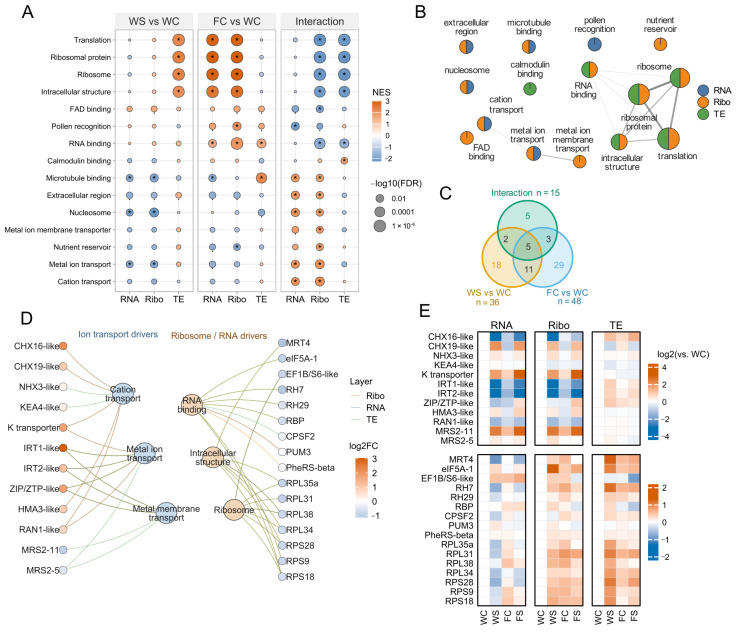
Genotype-dependent salt-responsive processes in WT and *fer* tomato roots. (**A**) GSEA of the 15 GO terms with significant genotype-by-salt interaction effects, shown for the WS vs. WC, FC vs. WC, and genotype-by-salt interaction comparisons across the RNA-abundance, ribosome-occupancy, and translational-efficiency layers. Dot color indicates normalized enrichment score (NES). (**B**) Leading-edge overlap network of significant interaction-associated GO terms. Each node is a GO term from (**A**); an edge is drawn between two GO terms if they share leading-edge genes, and node sectors are colored to indicate which of the RNA-abundance, ribosome-occupancy, or translational efficiency layers showed a significant interaction signal for that term. (**C**) Overlap of significant GO terms across the WS vs. WC, FC vs. WC, and genotype-by-salt interaction comparisons. (**D**) Representative leading-edge genes from the two major interaction-associated modules in (**B**). Edge colors indicate RNA-abundance, ribosome-occupancy, or translational efficiency leading-edge connections; gene-node colors indicate ribosome-occupancy interaction log_2_FC for ion-transport genes and translational efficiency interaction log_2_FC for ribosome/translation-related genes. (**E**) Log_2_ ratios of RNA abundance, ribosome occupancy, and translational efficiency relative to WC for ion-transport (top) and ribosome/translation-related (bottom) leading-edge genes. In panel (**A**), an asterisk (*) indicates FDR <0.05 and a middle dot (·) indicates FDR <0.20; significant terms in panels (**B**,**C**) were defined by FDR <0.05.

To isolate these FER-associated salt-responsive processes, we ranked genes by the genotype-by-salt interaction score (the *fer* salt response minus the WT salt response) separately for RNA abundance, ribosome occupancy, and translational efficiency, and performed gene set enrichment analysis (GSEA); the full ranked gene list is provided as Supplementary Table S8. The full GSEA overview also includes the WS vs. WC and FC vs. WC pairwise effects for comparison with the interaction results (Supplementary Figures S5 and S6; Table S5). Focusing on the interaction term, 15 GO terms showed significant genotype-by-salt interaction effects (FDR <0.05; Figure 4A). Because these 15 terms could represent either coherent biological themes or disparate functional signals, we performed leading-edge overlap analysis to examine whether they clustered into shared functional groups; this analysis identified two major functional modules among the interaction-associated terms (Figure 4B). The ribosome/translation-related module, including structural constituents of the ribosome, ribosome, translation, and intracellular structure, showed significant negative interaction signals in the ribosome-occupancy and translational efficiency layers. Because this contrast represents the *fer* salt response minus the WT salt response, these negative normalized enrichment score (NES) values indicate that the ribosome-occupancy and translational efficiency responses of this module were attenuated in *fer*. The ion-transport module, including cation transport, metal ion transport, and metal ion transmembrane transporter activity, showed positive interaction signals mainly in the RNA-abundance and ribosome-occupancy layers, indicating stronger salt-induced responses of ion-transport genes in *fer* than in WT. These interaction-associated GO terms overlapped only partially with the significant terms from the WS vs. WC and FC vs. WC comparisons, indicating that the interaction analysis captured FER-associated salt responses that were not evident from the pairwise comparisons alone (Figure 4C).

To illustrate the gene-level patterns underlying these two modules, we examined representative leading-edge genes from each module (Figure 4D; Table S6). The ion-transport module included cation/proton exchangers as well as metal-, K^+^- and Mg^2+^-transport-related genes, whereas the ribosome/translation-related module comprised ribosomal proteins and factors associated with translation, ribosome biogenesis/assembly, and RNA metabolism. For these genes, we compared log_2_ ratios of RNA abundance, ribosome occupancy, and translational efficiency relative to the WC baseline across the WS, FC, and FS conditions (Figure 4E), thereby visualizing how their profiles varied across genotype and treatment conditions. Ion-transport genes showed gene-specific RNA-abundance and ribosome-occupancy profiles, with many exhibiting stronger salt-associated changes in *fer* than in WT, in line with the module-level positive interaction signal. Ribosome/translation-related genes showed weaker salt-induced translational efficiency increases in *fer* than in WT, in line with the module-level negative interaction signal. Together, these gene-level profiles illustrate representative gene behaviors within the ion-transport and ribosome/translation-related interaction modules identified in tomato root salt responses.

### 2.5. Ribosome Occupancy in 5′ Leaders and uORF-Associated Translation Across Salt and Genotype Conditions

**Figure 5:**
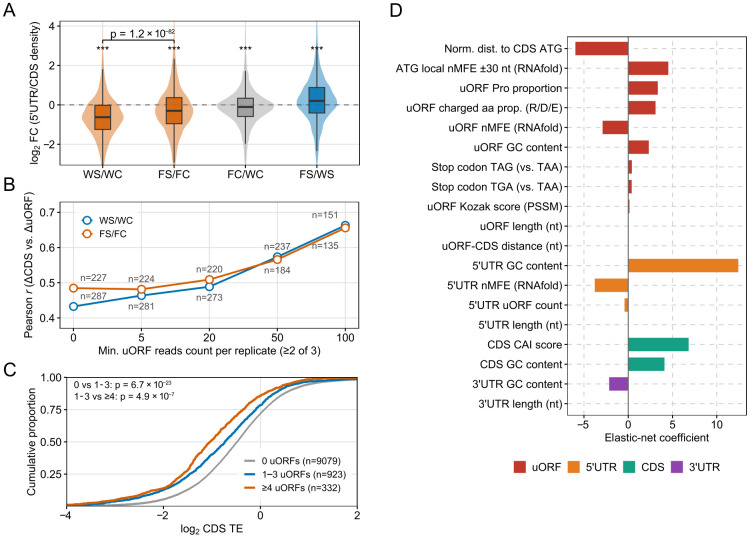
Ribosome occupancy in 5′UTRs and uORF-associated translation in tomato roots. (**A**) Genome-wide changes in relative 5′UTR ribosome occupancy, calculated as the log_2_FC of the length-normalized 5′UTR-to-CDS ribosome-density ratio, across salt-response and genotype comparison pairwise contrasts. (**B**) Coupling between ΔCDS translational efficiency and aggregate uORF translational efficiency change during salt treatment, measured by Pearson correlation across genes with RPF-supported translated uORFs in both compared conditions at different minimum uORF read-count thresholds. (**C**) Condition-level association between RPF-supported uORF number and CDS translational efficiency: cumulative CDS translational efficiency distributions for genes grouped by the number of RPF-supported uORFs detected by RiboBA. Genes were matched for CDS ribosome RPKM within each condition, and matched sets were pooled across the four conditions. (**D**) Sequence and transcript features distinguishing translated from matched non-translated ATG-uORFs, ranked by multivariate elastic-net logistic regression coefficients. Statistics: *** p<0.001 in panel (**A**), one-sample Wilcoxon tests with Benjamini–Hochberg (BH) correction; bracket in panel (**A**), two-sided Wilcoxon rank-sum test; *p* values in panel (**C**), pairwise Wilcoxon rank-sum tests with BH correction.

Among the ribosome/translation-related leading-edge genes, eIF5A-1 showed a strong WT salt-induced translational efficiency increase that was weaker in *fer* (Figure 4E). Given the reported roles of eIF5A in ribosome-pausing control and start-codon selection fidelity [41], we examined genome-wide ribosome allocation within 5′ leaders using the length-normalized 5′UTR-to-CDS ribosome density ratio (Figure 5A). This ratio decreased significantly under WT salt stress (WS vs. WC; median log_2_FC =−0.626, adjusted p<0.001), representing an approximately 6-fold larger shift than the baseline genotype difference (FC vs. WC; median log_2_FC =−0.105). Moreover, the salt-induced decrease was weaker in *fer* than in WT, as the FS vs. FC shift (median log_2_FC =−0.302) was approximately 2-fold smaller in magnitude than the WS vs. WC shift ($p=1.2\times 10^{-82}$). This genome-wide ribosome reallocation showed a weak but significant association with translational efficiency variation across all four comparisons (Spearman ρ=−0.084 to −0.119, all $p<10^{-13}$; Supplementary Figure S9A–D), consistent with a weak inhibitory relationship in which relatively greater 5′UTR ribosome occupancy is associated with lower CDS translational efficiency.

Because 5′ leader ribosome allocation may reflect uORF-associated translation, we examined uORF–CDS coupling genome-wide during the salt response. Among genes with RPF-supported translated uORFs detected in both compared conditions (Table S7, n=297 in WS vs. WC and n=236 in FS vs. FC), changes in CDS translational efficiency and aggregate uORF translational efficiency (ΔCDS TE and aggregate uORF ΔTE; “aggregate” denotes P-site signal summed across all translated uORFs within a gene before calculating translational efficiency, since a gene may carry more than one translated uORF; Methods 5.7) were positively correlated, and this coupling strengthened with higher minimum uORF read-count thresholds (Figure 5B), consistent with coordinated rather than predominantly competitive regulation of uORF and CDS translation during salt treatment. Beyond this dynamic coupling, we asked whether the static presence and number of translated uORFs were also associated with CDS translational efficiency. After matching genes for CDS ribosome RPKM, genes carrying 1–3 RPF-supported translated uORFs had significantly lower CDS translational efficiency than genes with no detected uORFs (median log_2_TE −0.734 vs. −0.482; approximately 1.2-fold lower; $p=6.7\times 10^{-23}$), with a further approximately 1.2-fold reduction for genes with ≥4 uORFs (median log_2_TE −1.038; $p=4.9\times 10^{-7}$; Figure 5C). Within the genome-wide set of genes with translated uORFs, the 5′UTR-to-CDS ratio and translational efficiency were anti-correlated more strongly than in the genome-wide background (Spearman ρ=−0.14 to −0.29 vs. −0.084 to −0.119; Supplementary Figure S9E), suggesting a stronger link between 5′ leader ribosome allocation and CDS translational efficiency in genes undergoing active uORF translation. Neither of these relationships was specific to the 29-gene ribosome/translation-associated buffered module: the module’s 5′UTR-to-CDS ratio–translational efficiency relationship did not differ from the genome-wide background (all p>0.28), and the module was not enriched for translated uORFs relative to the genome-wide rate (3 of 29 genes, 10.3%, vs. 5.6% genome-wide; Fisher’s exact test p=0.22).

Finally, we used elastic-net logistic regression on uORF-, UTR-, and CDS-level sequence features (Supplementary Figure S7) to identify predictors distinguishing translated from non-translated uORFs (Figure 5D). The model showed high predictive performance (cross-validated AUC = 0.931), and the strongest predictors included 5′UTR GC content, CDS codon adaptation index (CAI), local ATG-window folding features, CDS and uORF GC content, uORF amino-acid composition, and distance to the CDS ATG. These results suggest that translated uORFs are distinguished by both uORF-intrinsic features and broader transcript context, including region-specific folding properties.

## 3. Discussion

Transcript abundance alone provides an incomplete picture of plant stress responses, because translational regulation adds a further layer of control over protein output [13]. Ribo-seq studies of stress responses in Arabidopsis (hypoxia) [42], maize (drought) [43], rice (salinity) [15], and potato (drought/heat) [16] have shown that RNA abundance and ribosome loading are often only partially coupled, such that changes in transcript levels do not consistently predict corresponding changes in ribosome occupancy or protein-synthesis output. Meanwhile, FER has emerged as a receptor kinase linking salt responses with cell-wall integrity, Ca^2+^ signaling and osmotic-stress sensing [26,44], and has also been linked to translation-related pathways through RALF1–FER phosphorylation of eIF4E1 [28]. Whether this translation-related signaling capacity of FER extends to broader translational remodeling under salt stress, however, has not been examined. By integrating RNA-seq and Ribo-seq in WT and *fer* tomato roots, this study resolved FER-associated salt responses across transcript abundance, ribosome occupancy and translational efficiency layers. The results show that FER loss-of-function shifts ribosome/translation-associated genes toward elevated basal transcript abundance and ribosome occupancy, attenuates their salt-induced translational efficiency response, and is associated with altered 5′UTR-to-CDS ribosome allocation under salt stress.

### 3.1. The WT Salt Response Is Transcript-Dominant but Selectively Buffers Ribosome-Related Genes

In WT roots, the salt response was transcript-dominant, with Forwarded genes forming the largest regulatory class. The main translational exception was the ribosome/translation-enriched Buffered-down subset (Figure 2B–D): these genes showed salt-induced RNA decreases but concurrent increases in translational efficiency that more than compensated, yielding a net increase in ribosome occupancy. Notably, these buffered genes encode components of the protein-synthesis machinery itself. Most ribosome/translation-associated genes, consistent with their common characterization as housekeeping genes, showed no WS vs. WC differential expression (Figure 2D); the transcriptional repression observed here was not a general feature of this gene category, but was specific to this particular Buffered-down subset. Consistent with these results, a recent large-scale Ribo-seq/RNA-seq study in mammals showed that translationally buffered genes are enriched for ribosomal proteins and RNA-binding proteins [45]. Our data extend this buffering phenomenon to a plant abiotic-stress setting.

### 3.2. Loss-of-Function Fer Mutant Raises Basal Ribosome/Translation Gene Expression but Weakens Salt-Induced Translational Buffering

Comparison of untreated WT and *fer* roots (FC vs. WC) showed that FER loss-of-function reshaped the basal transcriptional and translational state before salt treatment. Most classified genes in this basal genotype comparison belonged to the Forwarded class, indicating that the basal effect of FER loss-of-function mainly involved coordinated changes in transcript abundance and ribosome occupancy rather than broad translational efficiency remodeling. Specifically, ribosome/translation-associated genes showed higher RNA abundance and ribosome occupancy in *fer*, whereas genes associated with stress, redox, and transport functions showed generally lower RNA abundance and ribosome occupancy. Thus, *fer* roots displayed a reshaped basal expression state, with ribosome/translation-associated genes already elevated and some stress-related functions relatively reduced.

This basal shift overlapped substantially with the Buffered-down ribosome/translation module in the WT salt response, with 18 of the 29 module genes already showing elevated ribosome occupancy in untreated *fer* roots. However, this elevated baseline does not appear to fully explain the module’s attenuated buffering: after correcting for each genotype’s baseline–response trend, the module’s excess translational efficiency response remained significantly greater in WT than in *fer*, and the genotype translational efficiency difference for this module reversed direction between control and salt conditions rather than persisting as would be expected under a baseline-only explanation. These results suggest that FER loss-of-function may affect this module in two distinguishable ways: by raising its basal expression state before stress exposure, and by separately contributing to the weakened salt-induced buffering response.

This module-level attenuation was mirrored at the broader functional-response level. Genotype-by-salt interaction analysis showed attenuated ribosome-occupancy and translational efficiency responses for ribosome/translation-related GO terms in *fer*, alongside stronger RNA-abundance and ribosome-occupancy responses for ion-transport terms (Figure 4). These stronger ion-transport responses do not necessarily indicate improved salt tolerance [26,27], but instead more likely reflect compensatory or dysregulated stress responses in *fer* roots.

In parallel, the 5′UTR-to-CDS analysis identified a broader, genome-wide FER-associated feature of ribosome allocation. The length-normalized 5′UTR-to-CDS ribosome density ratio shifted downward overall under WT salt stress, but this shift was weaker in *fer* (Figure 5A), suggesting that *fer* may affect the salt-induced redistribution of ribosomes from the 5′ leader toward the CDS. Among the ribosome/translation-related leading-edge genes, *eIF5A-1* provides a tentative clue to this leader-associated change, because its WT salt-induced translational efficiency increase was attenuated in *fer*, and eIF5A has been reported to participate in ribosome-pausing control and start-codon selection fidelity [41]. To our knowledge, this provides initial evidence for a potential connection between FER signaling and eIF5A-associated translational regulation; however, whether FER signaling directly controls *eIF5A-1* translational efficiency, and whether *eIF5A-1* contributes to the altered 5′UTR-to-CDS ribosome allocation observed in *fer*, remain to be determined.

### 3.3. uORF–CDS Translational Dynamics During the Salt Response Are Coordinated Rather than Predominantly Competitive

The uORF analyses revealed two patterns. First, genes with more RPF-supported translated uORFs showed lower CDS translational efficiency even after matching for CDS ribosome RPKM (Figure 5C), consistent with the established view that uORFs can reduce downstream main-ORF translation through impaired scanning or ribosome stalling [30,35]. Second, during the salt response, the aggregate uORF translational efficiency change and ΔCDS translational efficiency were positively correlated across genes with RPF-supported translated uORFs detected in both compared conditions, and this correlation increased with higher minimum uORF read-count thresholds (Figure 5B). Despite the overall repressive effect of uORFs on CDS translation, similar positive associations between uORF translational efficiency and CDS translational efficiency have been observed in vertebrate Ribo-seq analyses [46]. In our salt-response data, this positive coupling suggests that dynamic changes in uORF and CDS translation may primarily reflect changes in ribosome recruitment rather than direct uORF-mediated repression of downstream CDS translation.

### 3.4. Sequence and Transcript Features Distinguish Translated uORFs

Single-feature comparisons are useful for screening candidate features associated with translation, but they are limited because sequence and transcript properties are often interrelated [43]. The multivariate feature model therefore provides a more appropriate framework for evaluating features jointly associated with uORF translation (Figure 5D).

Several predictors were consistent with established models of eukaryotic 5′ leader translation. Stronger Kozak context scores were positively associated with uORF translation, consistent with the role of start-codon context in initiation-site selection [47]. The positive coefficient for nMFE around the uORF ATG suggests that weaker predicted secondary structure around the uORF start codon favors uORF initiation. The coefficient for normalized distance to the CDS was negative, indicating that translated uORFs tended to be closer to the main ORF; this is consistent with previous work showing that uORF–CDS spacing modulates downstream translational output [30,46]. More broadly, sequence features such as GC content and length have been linked to translational output in plant Ribo-seq studies [43].

Beyond these established features, the model highlighted three aspects that are not readily apparent from existing frameworks. First, RNAfold-derived nMFE features showed region-specific effects: weaker predicted structure near the uORF start codon favored translation, whereas nMFE values for the broader 5′UTR and uORF body showed negative coefficients, indicating that translated uORFs were associated with more stable predicted structures outside the immediate start-codon window. Thus, RNA structure showed region-specific effects rather than a uniform inhibitory relationship. Second, 5′UTR GC content and CDS CAI were the two largest positive predictors overall, suggesting that broader transcript sequence context contributes to whether a uORF is translated [43,48]. This was particularly informative for CDS CAI, which showed little univariate separation between translated and non-translated uORFs (BH-adjusted p=0.207) but became a strong positive predictor after joint modeling, underscoring the need for a multivariate framework. Third, translated uORFs showed higher proline and charged amino acid proportions, suggesting a contribution from uORF-intrinsic peptide features. These amino-acid patterns are reminiscent of plant conserved peptide uORFs that can mediate ribosome stalling or regulatory interactions through their encoded nascent peptides [30,33]. Together, these results indicate that RPF-supported uORF translation in tomato reflects a combination of broader transcript sequence context, start-codon environment, region-specific RNA structure, and uORF-encoded peptide composition.

### 3.5. Limitations

This study relies on a single CRISPR/Cas9-generated *fer* knockout line (*fer#2*), whose target-site edit and phenotypic characterization (root growth, lignin accumulation, pathogen-resistance phenotypes) were previously reported [24]. That study also provides independent genetic evidence that these phenotypes reflect specific disruption of FER signaling rather than an off-target artifact: an independently generated *ralf2* mutant, in the gene encoding the FER ligand, phenocopies *fer#2*, and mutating the downstream target gene *DIR19* in either background reverses the phenotypes, consistent with a defined RALF2-FER-MYB63-DIR19 pathway. However, no independent allele or genetic complementation was available for this sequencing dataset specifically, and no genome-wide off-target screen has been performed. Consequently, off-target or background mutations cannot be fully excluded, and the FER-associated effects reported here should be interpreted cautiously pending confirmation in an independent allele or complemented line. In addition, the mechanistic links proposed here, including the potential FER–eIF5A-1 connection and the proposed contribution of FER to translational buffering, are supported by correlative genomic evidence rather than direct functional validation. Such experiments were beyond the scope of this study and represent an important direction for future work.

## 4. Materials and Methods

### 4.1. Plant Materials and Salt Treatment

Tomato (*Solanum lycopersicum* L., cv. Condine Red, CR) was used as the wild-type (WT). The CRISPR/Cas9-generated *fer#2* mutant line (*fer*) in the CR background has been described previously [24].

Seedlings were grown in a greenhouse under controlled conditions with a 16 h light/8 h dark photoperiod at 25 °C during the light period and 21 °C during the dark period. Seedlings were maintained in 1/4-strength Hoagland’s medium before treatment. At 3–4 weeks after sowing, seedlings were transferred to 1/4-strength Hoagland’s medium containing 150 mM NaCl and treated for 5 days, whereas control seedlings were maintained in the same medium without NaCl. At the end of the treatment, root tissue was harvested from each group, immediately frozen in liquid nitrogen, and stored at −80 °C until use. Three independent biological replicates were collected for each condition, and each biological replicate consisted of pooled roots from five seedlings. The collected samples were used for matched RNA sequencing (RNA-seq) and ribosome profiling (Ribo-seq) library preparation.

### 4.2. RNA-Seq and Ribosome Profiling Library Preparation

Total RNA was extracted from tomato roots using the RNAprep Pure Plant Kit (DP419; Tiangen Biotech, Shanghai, China) according to the manufacturer’s instructions. RNA-seq libraries were then constructed using a standard RNA-seq library preparation workflow and sequenced by Guangzhou Gene Denovo Biotechnology Co., Ltd. (Guangzhou, China) on an Illumina sequencing platform in paired-end mode with a read length of 2 × 150 nt.

Ribosome profiling libraries were prepared using a modified plant Ribo-seq workflow. Briefly, frozen root tissue was ground in liquid nitrogen and lysed in ice-cold polysome extraction buffer supplemented with cycloheximide to arrest translating ribosomes. Lysates were clarified by centrifugation (17,000× *g*, 4 °C, 10 min). Clarified lysates were digested with RNase I and DNase I at room temperature for 45 min to generate ribosome-protected fragments (RPFs), and ribosome-associated fractions were recovered by size-exclusion chromatography using a MicroSpin S-400 column (GE27514001; Illumina, Inc., San Diego, CA, USA) equilibrated with polysome buffer. RPFs were then recovered by acid-phenol/chloroform extraction and column purification, followed by rRNA depletion (Ribo-Zero rRNA Removal Kit, Illumina, Inc., San Diego, CA, USA) and bead-based RNA cleanup. The purified fragments were 5′ phosphorylated with T4 polynucleotide kinase, sequentially ligated to 3′ and 5′ adaptors, reverse transcribed, and PCR amplified for 12 cycles (NEBNext Small RNA Library Prep Set for Illumina, New England Biolabs, Ipswich, MA, USA, catalog no. E7300). The final libraries were size-selected by PAGE, and fragments of approximately 140 bp were recovered and sequenced by Guangzhou Gene Denovo Biotechnology Co., Ltd. (Guangzhou, China) on an Illumina sequencing platform in paired-end mode, generating raw FASTQ files for each biological replicate.

### 4.3. Processing, Alignment, and Quantification of RNA-Seq and Ribo-Seq Data

Raw Ribo-seq reads were processed using fastp (v0.20.1) [49] with paired-end read merging and adapter correction enabled. Merged reads of 25–40 nt were retained. rRNA-derived reads were removed by aligning to tomato rRNA sequences with Bowtie (v1.3.1) [50] using the parameter -v 0. Non-rRNA reads were mapped to the tomato SL4.0 genome with ITAG4.0 annotation using STAR (v2.7.10b) [51] with –alignEndsType EndToEnd –outFilterMismatchNmax 1 –quantMode TranscriptomeSAM. Ribo-seq mapping efficiency was calculated as the number of genome-mapped reads divided by the number of input reads after rRNA removal.

Gene-level ribosome occupancy was quantified from CDS-overlapping Ribo-seq reads using featureCounts in the Rsubread package (v2.22.1) [52]. RNA-seq reads were aligned to the same reference genome using STAR in two-pass mode, and gene-level RNA-seq counts were obtained with featureCounts. Multi-mapping and multi-overlap reads were excluded. For sample-level visualization, raw count matrices were TMM-normalized and transformed to logCPM using edgeR (v4.6.3) [53].

### 4.4. Reproducibility, PCA, and Ribo-Seq Quality Control

For reproducibility analysis, genes with counts ≥10 in at least three samples were retained. Sample reproducibility was assessed using squared Pearson correlation coefficients calculated from TMM-normalized logCPM values. Principal component analysis was performed on the same filtered, TMM-normalized RNA-seq and Ribo-seq gene-level matrices.

Ribo-seq P-site calibration and quality control analyses were performed with riboWaltz (v2.0) [54]. Transcriptome-aligned BAM files were filtered to retain uniquely mapped reads and converted to riboWaltz-compatible input using the ITAG4.0 annotation. P-site offsets were inferred automatically and used to summarize RPF length distribution, CDS reading-frame periodicity, read distribution across 5′UTR, CDS, and 3′UTR regions, and metagene profiles around annotated start and stop codons.

### 4.5. Differential Expression, Translational Efficiency, and Regulatory Classification

Differential transcript-abundance and ribosome-occupancy analyses were performed with DESeq2 (v1.48.2) [55]. Gene-level translational efficiency was defined as normalized ribosome occupancy divided by normalized RNA abundance following the deltaTE framework [14]. Translational regulatory classes were assigned as follows: Forwarded genes showed significant transcript-abundance and ribosome-occupancy changes without significant translational efficiency changes; Buffered genes showed transcript-abundance and translational efficiency changes in opposite directions; Intensified genes showed transcript-abundance and translational efficiency changes in the same direction; and Exclusive genes showed significant ribosome-occupancy and translational efficiency changes without significant transcript-abundance changes. For directional summaries, Forwarded, Buffered, and Intensified classes were split by RNA log_2_FC direction, whereas Exclusive genes were split by ribosome-occupancy log_2_FC direction.

For genes with defined ΔRNA and ΔRibo values, excluding genes for which ΔRNA=0, a per-gene buffering index was calculated as 1−(ΔRibo/ΔRNA). ΔRNA and ΔRibo were calculated from condition-mean log_2_CPM values averaged across biological replicates for RNA abundance and ribosome occupancy (WS vs. WC in WT; FS vs. FC in *fer*). To assess whether genotype-dependent translational efficiency responses could be explained by baseline translational efficiency differences alone, we used a split-half, leave-one-out design: for each genotype, one of the three biological replicates was held out to estimate baseline translational efficiency (control condition), and the salt-induced ΔTE was estimated independently from the remaining two replicates only, so that baseline and response estimates never shared a replicate. This procedure was repeated over all three leave-one-out folds and averaged per gene. A background baseline-versus-response trend was fitted across all genes, and each gene’s residual from this trend was calculated. Residuals for tested gene sets were compared against zero using a permutation test (10,000 resamples), and differences between WT and *fer* residuals were assessed using a Mann–Whitney U test.

### 4.6. GO Enrichment and Gene Set Enrichment Analysis

GO over-representation analysis was performed with clusterProfiler (v4.16.0) [56] using ITAG4.0-derived GO annotations. The background was defined as the set of genes entering the corresponding deltaTE analysis.

Gene set enrichment analysis (GSEA) was performed with fgsea (v1.34.2) [57]. For genotype-by-salt interaction analysis, genes were ranked using the genotype-by-salt interaction coefficient from a DESeq2 model with the design ~ genotype + treatment + genotype:treatment, corresponding to $\log_{2}\left(\mathrm{FS}/\mathrm{FC}\right)-\log_{2}\left(\mathrm{WS}/\mathrm{WC}\right)$. This interaction coefficient was calculated separately for RNA abundance, ribosome occupancy, and translational efficiency. GO terms with 10–500 genes were retained, and fgsea was run with nPermSimple = 10,000. Leading-edge genes from GO terms passing the GSEA FDR threshold were used to construct the GO-term overlap network and candidate-gene visualizations.

### 4.7. uORF Identification and Leader-Associated Translation Analysis

Sequence-defined uORFs and RPF-supported translated uORFs were identified using RiboBA [38] from ITAG4.0 transcript annotation and adapter-trimmed Ribo-seq reads. RPF-supported uORF events were collapsed by identical transcript-coordinate intervals, defined by transcript, strand, uORF start, and uORF end. For translated-uORF feature modeling, translated uORFs were defined as uORFs with the same transcript-coordinate interval detected in at least two biological replicates in any of the four conditions.

For relative 5′UTR ribosome occupancy, RiboBA-derived P-site counts were summed separately within 5′UTR and CDS regions and divided by region length to calculate P-site density. The length-normalized ratio was defined as 5′UTR P-site density divided by CDS P-site density. Gene-level condition means were calculated across three biological replicates, and log_2_ fold changes in this ratio were computed for the WT salt response (WS vs. WC), the *fer* salt response (FS vs. FC), the basal genotype comparison (FC vs. WC), and the genotype comparison under salt treatment (FS vs. WS). For visualization of the genome-wide relationship between this ratio and translational efficiency, genes were grouped into 20 quantile bins by the log_2_FC of this ratio, and bin means ± SEM were calculated (Supplementary Figure S9A–D); Spearman correlations were calculated on the unbinned, per-gene values. For the uORF-carrying gene subset (uORF+) analysis (Supplementary Figure S9E), Spearman correlations and their 95% confidence intervals were estimated by bootstrap resampling (2000 resamples with replacement; median and 2.5th/97.5th percentiles reported).

To examine uORF–CDS translational efficiency coupling, genes carrying RPF-supported translated uORFs detected in both conditions of a comparison were retained. Aggregate uORF P-site signal was calculated by summing P-site counts across uORF intervals within each gene and normalizing the summed counts to CPM. Aggregate uORF translational efficiency was then calculated by dividing aggregate uORF P-site CPM by RNA CPM. CDS translational efficiency was calculated analogously from CDS P-site CPM and RNA CPM. Pearson correlations between changes in aggregate uORF translational efficiency and CDS translational efficiency were calculated across a series of minimum uORF read-count thresholds.

For CDS translational efficiency cumulative distribution analysis, genes were grouped in each condition by the number of RPF-supported uORFs detected by RiboBA in at least one biological replicate: 0, 1–3, or ≥4 uORFs. To control for CDS ribosome abundance, genes in the 0 and 1–3 groups were matched to the ≥4 group using bins of mean CDS ribosome RPKM. Matched condition-specific gene sets were then combined across the four conditions.

### 4.8. Sequence-Feature Analysis and Elastic-Net Modeling of Translated uORFs

Sequence and transcript features were extracted for translated and non-translated ATG-uORFs from transcript sequences using in-house scripts. Non-translated uORFs were obtained by scanning 5′UTRs of matched control genes for ATG-initiated ORFs fully contained within the 5′UTR, excluding intervals overlapping translated uORFs. Features included uORF length, uORF–CDS distance, normalized distance to the CDS ATG, uORF and 5′UTR GC content, RNAfold-derived normalized minimum free energy (nMFE) [58] for the uORF, 5′UTR, and local uORF ATG window, uORF start-codon Kozak score, uORF amino-acid composition, 5′UTR uORF number, stop-codon type, and CDS CAI score. The Kozak score was calculated as a position-specific scoring matrix score from the nucleotide context around annotated CDS start codons and then applied to uORF start-codon contexts [47]. CAI was calculated according to the codon adaptation index framework [48].

Feature associations were modeled using elastic-net binomial logistic regression implemented in glmnet (v5.0) [59]. Predictors were median-imputed where necessary and standardized during model fitting. The model used α=0.5, with λ selected by 10-fold cross-validation using AUC as the optimization metric. Predictive performance was evaluated primarily by cross-validation, with an internal held-out test set used as an additional assessment, and non-zero coefficients at the minimum cross-validated λ were used to rank features distinguishing translated from non-translated uORFs.

### 4.9. Statistical Analysis

Unless otherwise stated, statistical tests were two-sided. Wet-weight differences between treatments and genotypes were assessed by Wilcoxon rank-sum tests. For transcript-abundance and ribosome-occupancy DEG summaries, differentially regulated genes were defined by adjusted p<0.05 and $|\log_{2}\mathrm{FC}|\geq 1$. For deltaTE regulatory classification, transcript-abundance, ribosome-occupancy, and translational efficiency components were considered significant at adjusted p<0.05.

For GO over-representation analysis, Benjamini–Hochberg-adjusted *p* values were used, with significance thresholds as indicated in the corresponding figures. For GSEA, FDR <0.05 was used as the significance threshold; FDR <0.20 is shown as a suggestive level in Figure 4A.

Gene-set comparisons used Wilcoxon rank-sum tests for unpaired groups or paired Wilcoxon signed-rank tests for matched-control comparisons. For Figure 5A, one-sample Wilcoxon signed-rank tests against zero were Benjamini–Hochberg corrected, whereas the comparison between WT and *fer* salt-response shifts used a Wilcoxon rank-sum test. CDS translational efficiency distributions grouped by uORF count were tested by pairwise Wilcoxon rank-sum tests with Benjamini–Hochberg correction. Translated versus non-translated uORF sequence-feature comparisons were tested by Wilcoxon rank-sum tests with Benjamini–Hochberg correction; stop-codon type was evaluated by chi-squared test.

For the split-half baseline-versus-response analysis, residuals of tested gene sets from the genome-wide background trend were tested against zero by permutation (10,000 resampled gene sets of the same size drawn from the background) and compared between genotypes using a Mann–Whitney U test. Associations between the 5′UTR-to-CDS ratio and translational efficiency were assessed by Spearman correlation across genes with data in both compared conditions, separately for genotype-dependent and salt-response contrasts.

## 5. Conclusions

This study provides a matched transcriptome–translatome dissection of FER-associated salt responses in tomato roots. In WT roots, salt stress was dominated by transcript-driven remodeling, but a ribosome/translation-associated gene module showed selective translational buffering, indicating that translational control refines the transcriptional response. FER loss-of-function shifted this module toward an elevated basal state and attenuated its salt-induced translational efficiency response, while strengthening ion-transport responses, consistent with a dysregulated stress state. Genome-wide analyses of ribosome allocation within the 5′ leader further showed that WT salt stress reduced relative 5′UTR ribosome occupancy, with this effect attenuated in *fer*, and that aggregate uORF and CDS translational efficiency changes were positively coupled during salt treatment. Feature modeling indicated that translated uORFs are distinguished by broader transcript context, start-codon environment, region-specific RNA structure, and uORF peptide composition. Together, these results support a model in which FER contributes to tomato root salt responses by preserving the capacity for selective translational control. We also identify FER signaling, *eIF5A-1*, and leader-associated ribosome allocation as candidate areas for future mechanistic study.

## Data Availability

RNA-seq and Ribo-seq data for wild-type and *fer* tomato roots under salt stress are available in ArrayExpress under E-MTAB-17154 and E-MTAB-17159. Figure-ready data and plotting code are available in Zenodo under DOI: https://doi.org/10.5281/zenodo.20563674.

## Supplementary Materials

The following supporting information can be downloaded at: https://www.mdpi.com/article/10.3390/plants15152278/s1, Figure S1: Quality assessment of RNA-seq and Ribo-seq libraries. Figure S2: RNA-abundance and ribosome-occupancy changes across salt-response and genotype comparisons. Figure S3: Relationship between RNA-abundance and translational-efficiency (TE) changes across salt and genotype comparisons. Figure S4: Gene Ontology enrichment of regulatory classes in *fer* salt-response and genotype comparisons. Figure S5: Gene set enrichment analysis (GSEA) overview of genotype, salt, and interaction effects across RNA-abundance, ribosome occupancy, and translational efficiency layers, Part 1. Figure S6: GSEA overview of genotype, salt, and interaction effects across RNA-abundance, ribosome occupancy, and translational efficiency layers, Part 2. Figure S7: Sequence and transcript features of translated and non-translated uORFs. Figure S8: Genome-wide baseline–response relationship and per-gene translational buffering metrics for the ribosome/translation module. Figure S9: Correlation between the 5′UTR-to-CDS ratio and translational efficiency across salt-response and genotype comparisons, and among uORF-carrying genes. Table S1: Alignment statistics for Ribo-seq libraries; Table S2: Alignment statistics for RNA-seq libraries; Table S3: Significant differential transcript abundance, ribosome occupancy, translational efficiency, and WS/WC regulatory class assignments; Table S4: Gene Ontology enrichment results for regulatory classes across salt and genotype comparisons; Table S5: Gene set enrichment analysis results for salt, genotype, and genotype-by-salt interaction effects; Table S6: Gene-level source tables for ribosome/translation-associated buffered modules and interaction candidate genes; Table S7: Transcript-coordinate translated uORFs detected in at least two replicates in any condition; Table S8: Genome-wide gene list ranked by the genotype-by-salt interaction score (the *fer* salt response minus the WT salt response) for RNA abundance, ribosome occupancy, and translational efficiency, underlying the GSEA shown in Figure 4A.

## Abbreviations

The following abbreviations are used in this manuscript:

AUC	area under the curve
BH	Benjamini–Hochberg
CAI	codon adaptation index
CDS	coding sequence
CK	control condition
CPM	counts per million
DEG	differentially expressed gene
FC	*fer* control
FDR	false discovery rate
FER	FERONIA
FS	*fer* salt
GO	Gene Ontology
GSEA	gene set enrichment analysis
logCPM	log counts per million
nMFE	normalized minimum free energy
NES	normalized enrichment score
PCA	principal component analysis
Ribo-seq	ribosome sequencing
RNA-seq	RNA sequencing
RPF	ribosome-protected fragment
RPKM	reads per kilobase per million mapped reads
S150	150 mM NaCl salt treatment
TE	translational efficiency
TMM	trimmed mean of M-values
uORF	upstream open reading frame
UTR	untranslated region
WC	WT control
WS	WT salt (150 mM NaCl)
WT	wild-type
