# Supplementary material for: FERONIA Modulates Translational Buffering Capacity of Ribosome-Associated Gene Module in Salt-Stressed Tomato Roots
Source: Plants (Basel). 2026 Jul 25;15(15):2278. doi: 10.3390/plants15152278 (PMC13467700; doi:10.3390/plants15152278)
Supplement: Supplementary file 1 [file plants-15-02278-s001.zip › supplementary_materials.pdf]

Supplementary Materials

FERONIA Modulates Translational Buffering Capacity of a Ribosome-Associated Gene Module in Salt-Stressed Tomato Roots

Junyu Bai[1,†], Yanfen Fan[2,3,4,†], Ruolin Yang[1,*] and Jingquan Yu[2,4,5,*]

[1]College of Life Sciences, Northwest A&F University, Yangling 712100, Shaanxi, China; bai.junyu.bio@gmail.com (J.B.); desert.ruolin@gmail.com (R.Y.)

[2]Department of Horticulture, Zhejiang University, Zijingang Campus, 866 Yuhangtang Road, Hangzhou 310058, China

[3]Department of Chemical and Biomolecular Engineering, University of Illinois at Urbana-Champaign, Urbana, IL 61801, USA; yanfen36@illinois.edu (Y.F.)

[4]College of Horticulture, Northwest Agriculture and Forestry University, Yangling 712100, Shaanxi, China

[5]Key Laboratory of Horticultural Plants Growth and Development, Agricultural Ministry of China, 866 Yuhangtang Road, Hangzhou 310058, China; jqyu@zju.edu.cn (J.Y.)

* Correspondence: desert.ruolin@gmail.com (R.Y.); jqyu@zju.edu.cn (J.Y.)

† These authors contributed equally to this work.

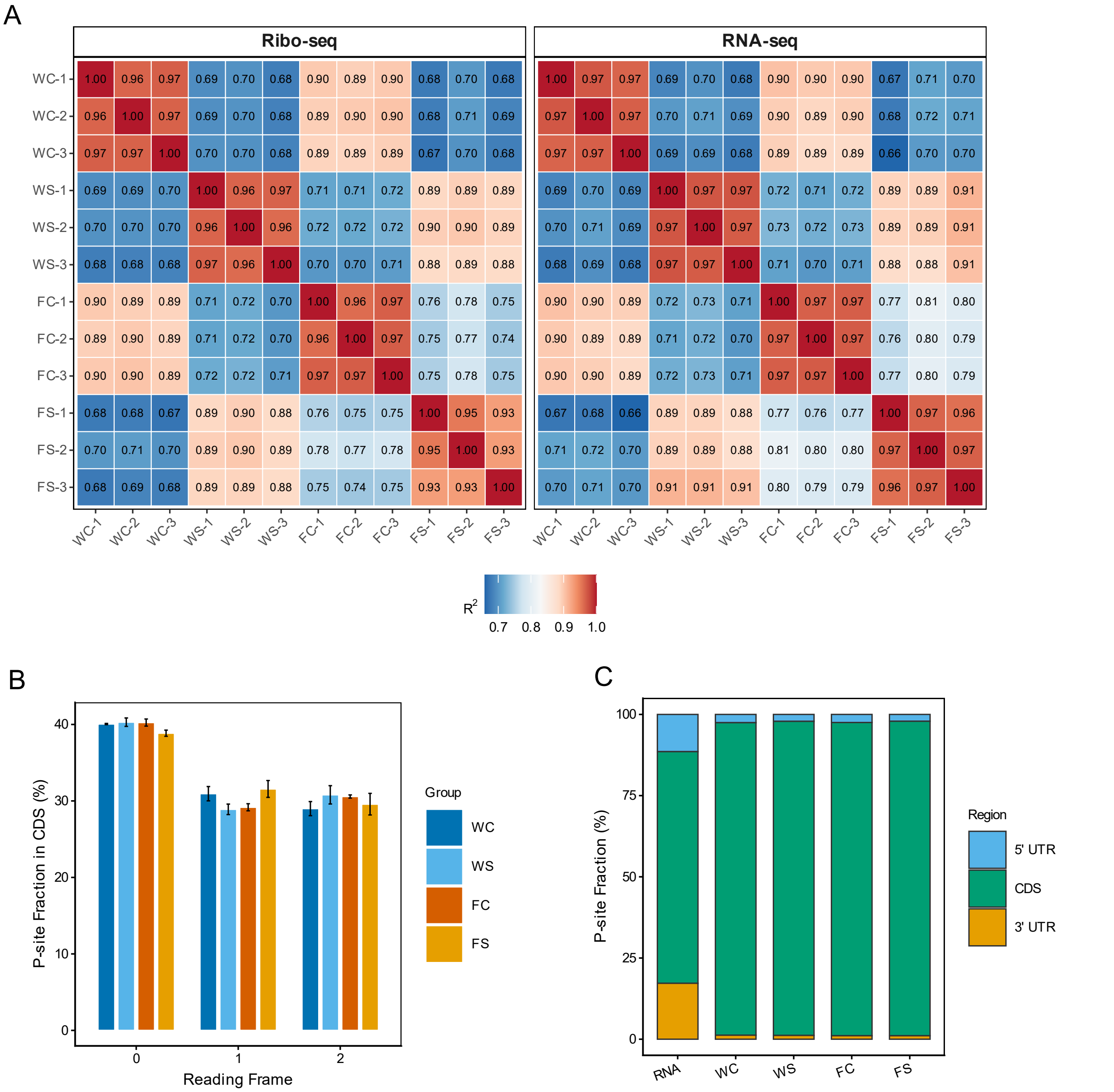

Supplementary Figure S1. Quality assessment of RNA-seq and Ribo-seq libraries. **(A)** Pairwise R^2 correlation heatmaps of RNA-seq and Ribo-seq libraries across 12 samples from WC, WS, FC, and FS conditions, with three biological replicates per condition. **(B)** Reading-frame distribution of CDS P-sites across the four experimental groups. **(C)** Regional distribution of Ribo-seq P-sites and RNA-seq reads across 5′UTR, CDS, and 3′UTR regions. Condition codes: WC, WT control; WS, WT salt; FC, *fer* control; FS, *fer* salt.

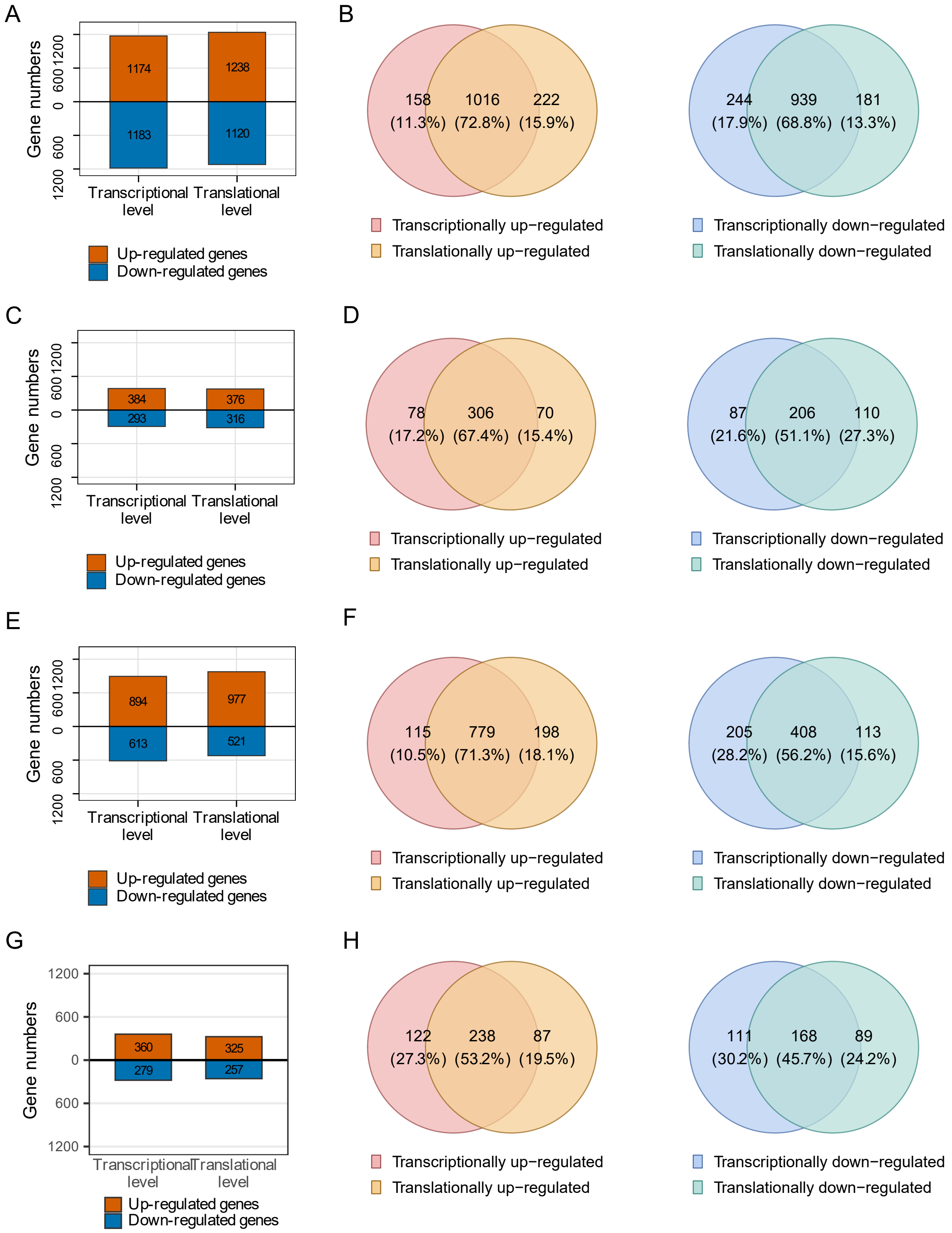
A
Gene numbers
1200
600
0
600
1200
1174
1238
1183
1120
Transcriptional level
Translational level
Up-regulated genes
Down-regulated genes
B
158 (11.3%)
1016 (72.8%)
222 (15.9%)
244 (17.9%)
939 (68.8%)
181 (13.3%)
Transcriptionally up−regulated
Translationally up−regulated
Transcriptionally down−regulated
Translationally down−regulated
C
Gene numbers
384
376
293
316
Transcriptional level
Translational level
Up-regulated genes
Down-regulated genes
D
78 (17.2%)
306 (67.4%)
70 (15.4%)
87 (21.6%)
206 (51.1%)
110 (27.3%)
Transcriptionally up−regulated
Translationally up−regulated
Transcriptionally down−regulated
Translationally down−regulated
E
Gene numbers
894
977
613
521
Transcriptional level
Translational level
Up-regulated genes
Down-regulated genes
F
115 (10.5%)
779 (71.3%)
198 (18.1%)
205 (28.2%)
408 (56.2%)
113 (15.6%)
Transcriptionally up−regulated
Translationally up−regulated
Transcriptionally down−regulated
Translationally down−regulated
G
Gene numbers
360
325
279
257
Transcriptional level
Translational level
Up-regulated genes
Down-regulated genes
H
122 (27.3%)
238 (53.2%)
87 (19.5%)
111 (30.2%)
168 (45.7%)
89 (24.2%)
Transcriptionally up−regulated
Translationally up−regulated
Transcriptionally down−regulated
Translationally down−regulated

Supplementary Figure S2. RNA-abundance and ribosome-occupancy changes across salt-response and genotype comparisons. (A, C, E, G) Numbers of up- and down-regulated genes at the RNA-abundance and ribosome-occupancy levels in the WS versus WC, FC versus WC, FS versus FC, and FS versus WS comparisons, respectively. **(B, D, F, H)** Overlap between RNA-abundance- and ribosome-occupancy-regulated genes for the corresponding up- and down-regulated subsets. Differentially regulated genes were defined by adjusted $P < 0.05$ and $|\log_2 \mathrm{FC}| \geq 1$.

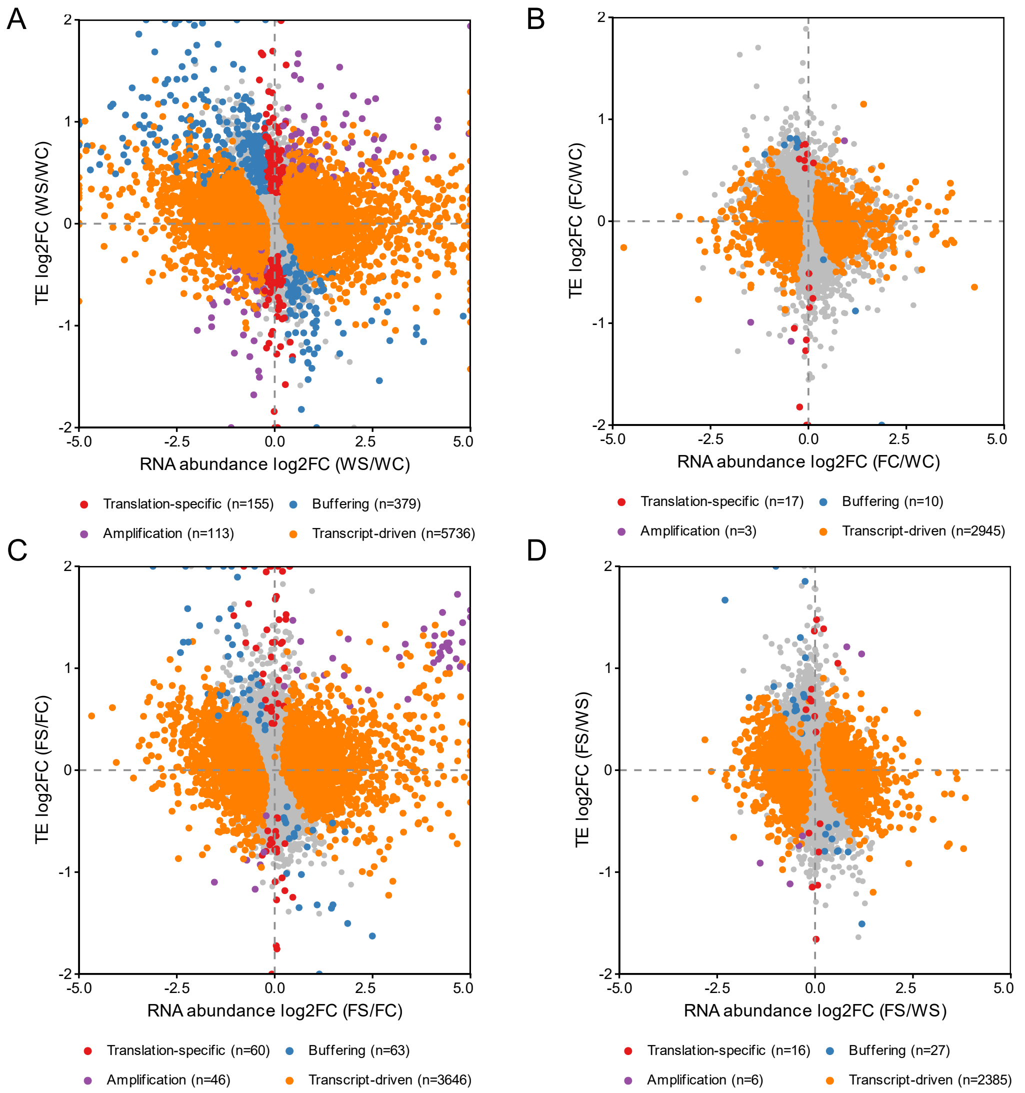

Supplementary Figure S3. Relationship between RNA-abundance and translational-efficiency (TE) changes across salt and genotype comparisons. (A–D) RNA abundance and translational efficiency changes in the WS versus WC, FC versus WC, FS versus FC, and FS versus WS comparisons, respectively. Genes were coloured according to regulatory classes defined by RNA-abundance, ribosome-occupancy, and translational efficiency changes. Class labels denote Forwarded (Transcript-driven), Buffered (Buffering), Intensified (Amplification), and Exclusive (Translation-specific), as defined in Figure 2.

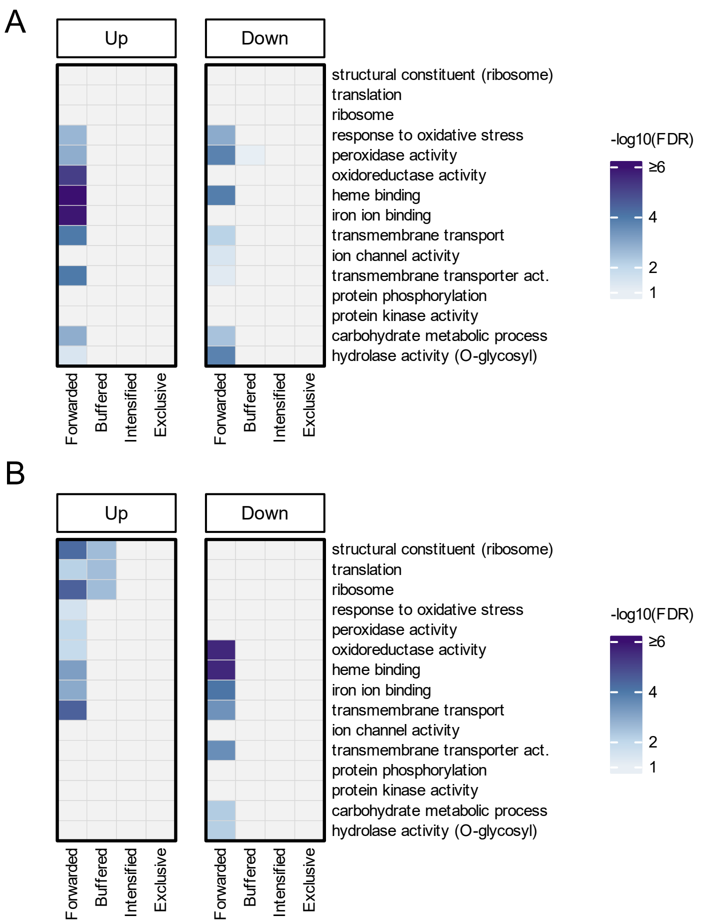

Supplementary Figure S4. Gene Ontology enrichment of regulatory classes in *fer* salt-response and genotype comparisons. (A) Gene Ontology enrichment of Forwarded, Buffered, Intensified, and Exclusive classes in the *fer* salt-response comparison (FS versus FC). **(B)** Gene Ontology enrichment in the genotype comparison under salt treatment (FS versus WS). For Forwarded, Buffered, and Intensified classes, up- and down-regulated subsets are defined by RNA $\log_2$FC direction; for the Exclusive class, by ribosome-occupancy $\log_2$FC direction. Coloured cells indicate significantly enriched terms (FDR < 0.10); colour intensity represents $-\log_{10}(\mathrm{FDR})$, capped at 6.

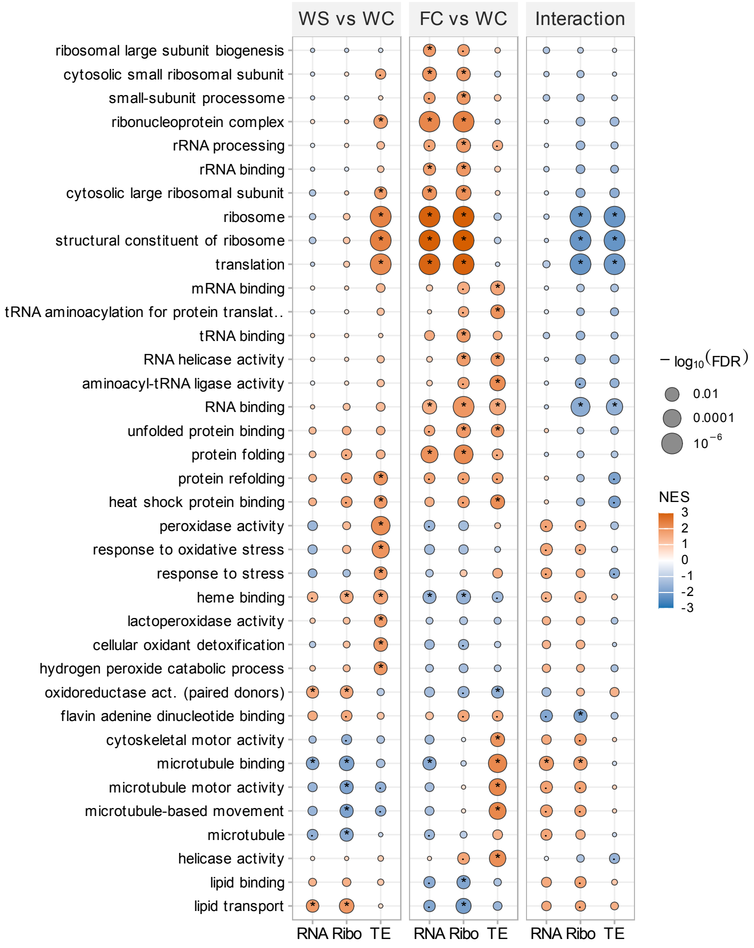

Supplementary Figure S5. Gene set enrichment analysis (GSEA) overview of genotype, salt, and interaction effects across RNA-abundance, ribosome-occupancy, and translational efficiency layers, Part 1. GSEA bubble plots showing all GO terms with FDR < 0.05 in at least one layer of at least one comparison, across the WT salt response (WS versus WC), basal genotype comparison (FC versus WC), and genotype-by-salt interaction effects in RNA-abundance, ribosome-occupancy, and translational efficiency layers. Bubble colour indicates normalised enrichment score (NES), and bubble size indicates $-\log_{10}(\mathrm{FDR})$. Asterisks indicate FDR < 0.05.

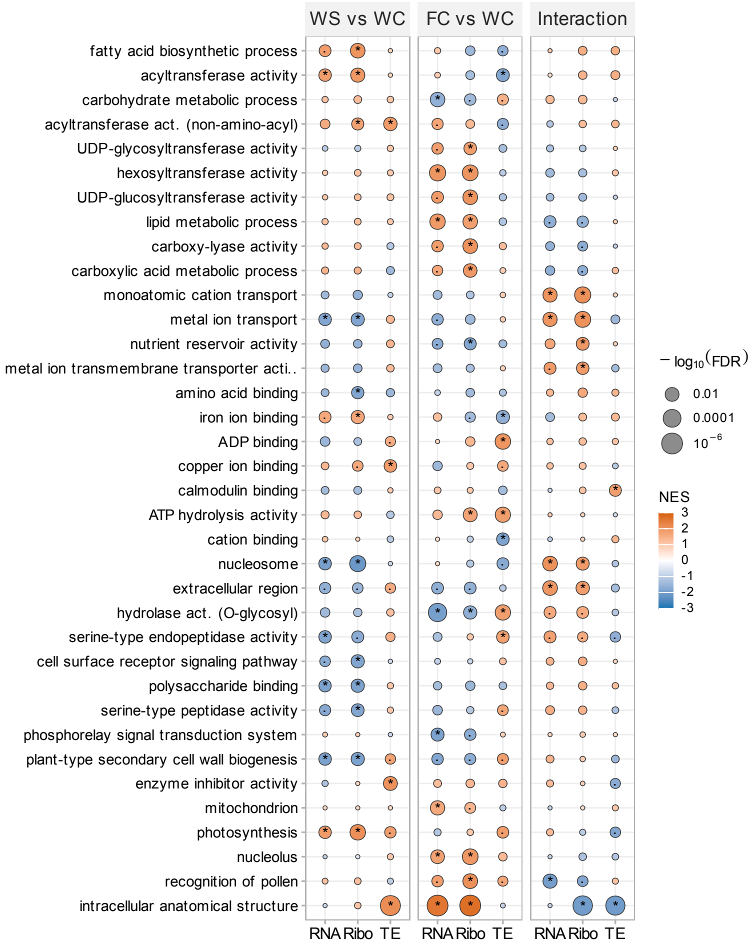

Supplementary Figure S6. GSEA overview of genotype, salt, and interaction effects across RNA-abundance, ribosome-occupancy, and translational efficiency layers, Part 2. Continuation of the GSEA overview shown in Supplementary Figure S5. Bubble colour indicates normalised enrichment score (NES), and bubble size indicates $-\log_{10}(\mathrm{FDR})$. Asterisks indicate FDR < 0.05.

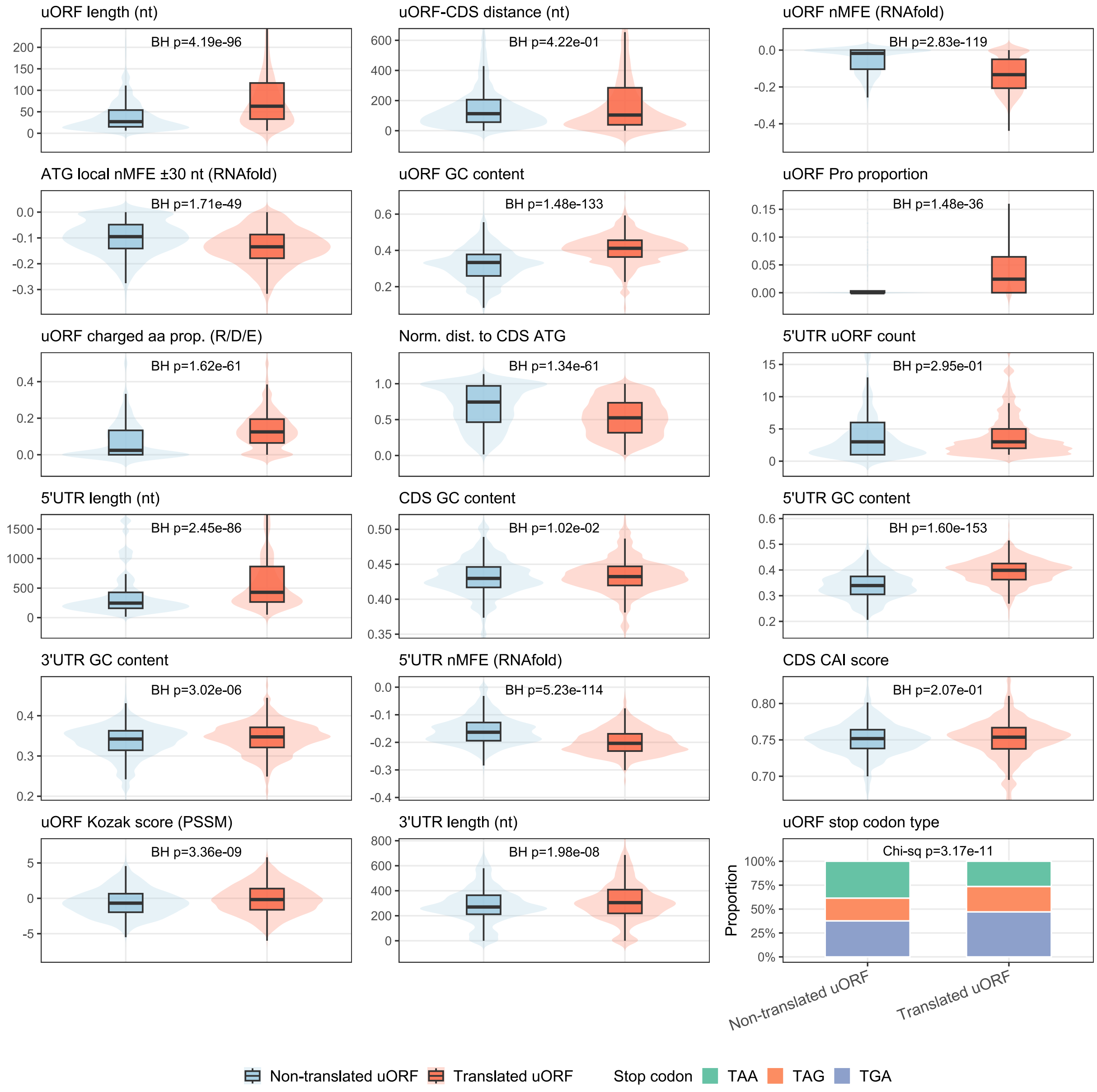

Supplementary Figure S7. Sequence and transcript features of translated and non-translated uORFs. Distributions of uORF, 5′UTR, and CDS sequence features compared between translated uORFs and non-translated ATG-uORFs identified in matched control genes. Translated uORFs were defined by detection of the same transcript-coordinate interval in at least two biological replicates in any of the four conditions. BH-adjusted P values are shown for Wilcoxon rank-sum tests, except for uORF stop-codon type, which was tested using a chi-squared test.

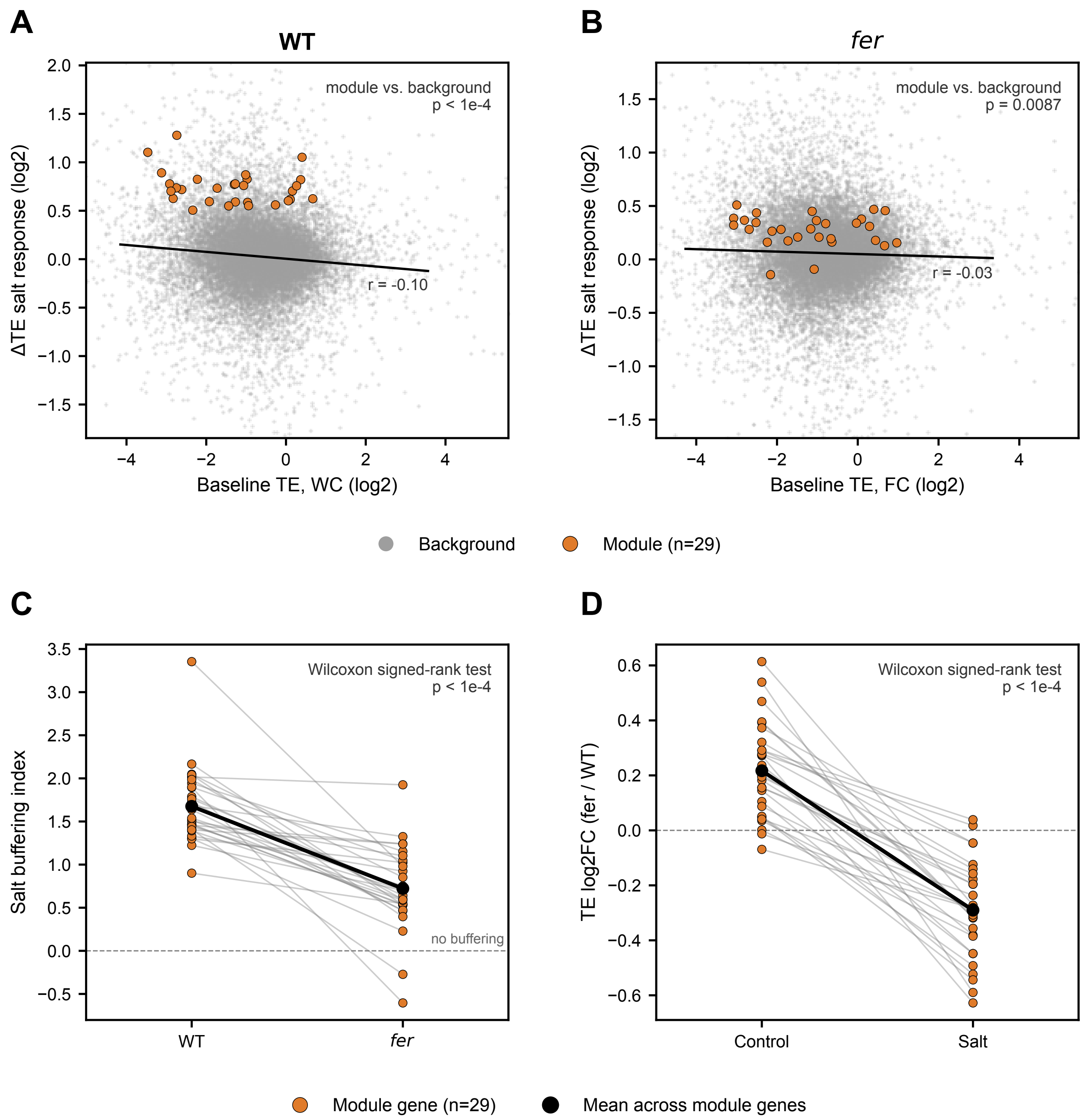

Supplementary Figure S8. Genome-wide baseline–response relationship and per-gene translational buffering metrics for the ribosome/translation module. (A, B) Baseline translational efficiency versus the salt-induced ΔTE response (split-half design), for background genes (grey, $n = 15{,}749$) and the 29-gene ribosome/translation Buffered-down module (orange). Black line, fitted background trend. Module residuals from the background trend were assessed by permutation test ($P < 1 \times 10^{-4}$ in WT, $P = 0.0087$ in *fer*); the residual was significantly larger in WT than in *fer* (Mann–Whitney U test, $U = 2$, $P < 0.0001$). **(C)** Per-gene salt buffering index [$1 - (\Delta\mathrm{Ribo}/\Delta\mathrm{RNA})$, S150 vs. CK] for the

29 module genes in WT and *fer* (paired Wilcoxon signed-rank test, $P < 1 \times 10^{-4}$). Index $= 0$ (dashed line), no buffering; index > 1, over-compensation. **(D)** TE $\log_2$FC (*fer*/WT) for the 29 module genes under control and salt conditions (paired Wilcoxon signed-rank test, $P < 1 \times 10^{-4}$, decreased in 29 of 29 genes; sign reversed, *fer* $>$ WT under control but *fer* $<$ WT under salt, in 25 of 29 genes).

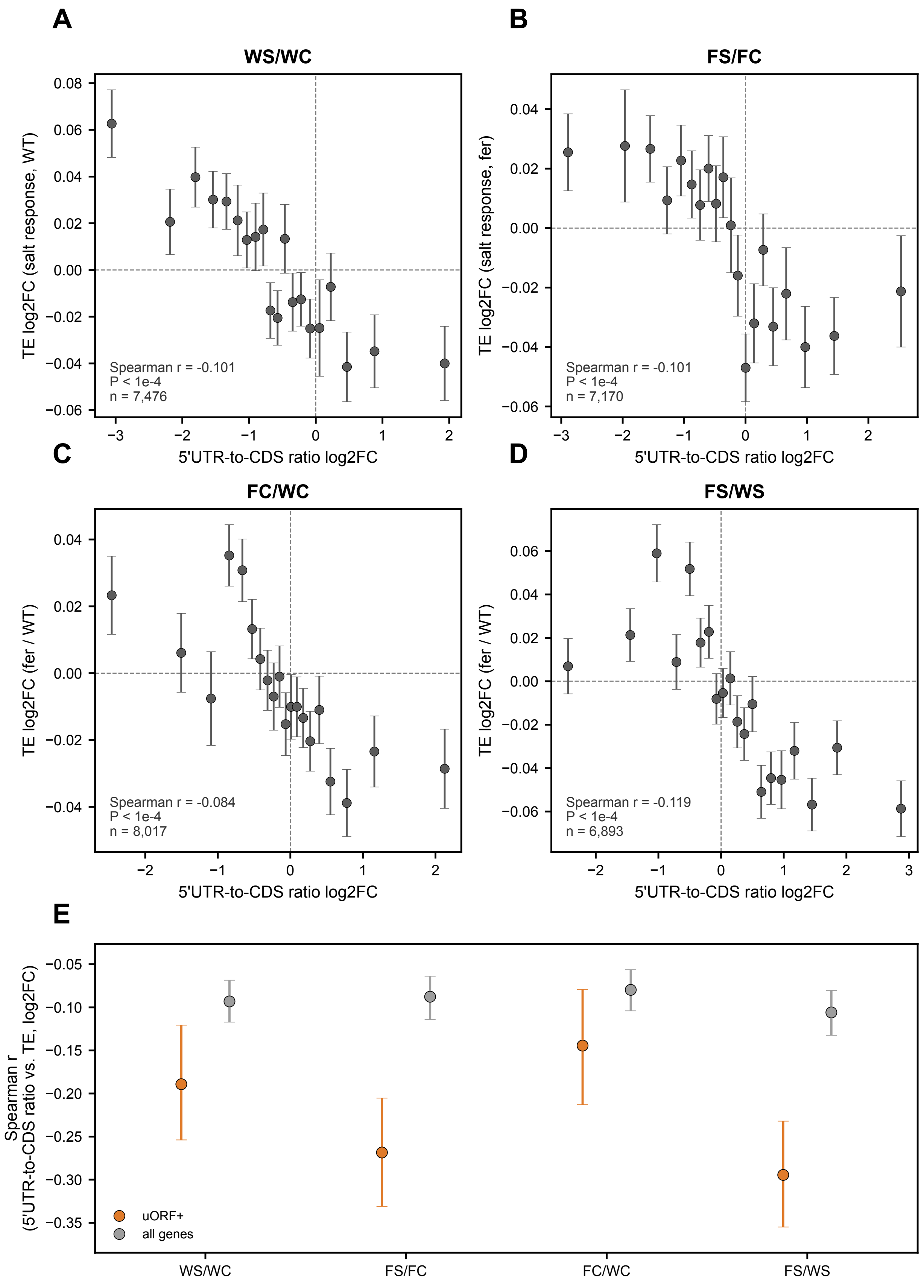
A
WS/WC
TE log2FC (salt response, WT)
Spearman r = -0.101
P < 1e-4
n = 7,476
5'UTR-to-CDS ratio log2FC
B
FS/FC
TE log2FC (salt response, fer)
Spearman r = -0.101
P < 1e-4
n = 7,170
5'UTR-to-CDS ratio log2FC
C
FC/WC
TE log2FC (fer / WT)
Spearman r = -0.084
P < 1e-4
n = 8,017
5'UTR-to-CDS ratio log2FC
D
FS/WS
TE log2FC (fer / WT)
Spearman r = -0.119
P < 1e-4
n = 6,893
5'UTR-to-CDS ratio log2FC
E
Spearman r
(5'UTR-to-CDS ratio vs. TE, log2FC)
uORF+
all genes
WS/WC
FS/FC
FC/WC
FS/WS

Supplementary Figure S9. Correlation between the 5′UTR-to-CDS ratio and translational efficiency across salt-response and genotype comparisons, and among uORF-carrying genes. (A–D) Change in the 5′UTR-to-CDS ratio ($\log_2$FC) versus change in translational efficiency ($\log_2$FC) across the same four contrasts as in Figure 5A: the WT salt response (A, WS/WC, $n = 7{,}476$), the *fer* salt response (B, FS/FC, $n = 7{,}170$), the basal genotype comparison (C, FC/WC, $n = 8{,}017$), and the genotype comparison under salt treatment (D, FS/WS, $n = 6{,}893$). **(E)** Spearman correlation (5′UTR-to-CDS ratio $\log_2$FC vs. TE $\log_2$FC) across the same four contrasts, for genes carrying an RPF-supported translated uORF (uORF+, $n = 881$; orange) alongside the genome-wide value already shown in (A–D) (all genes; grey), points show bootstrap median and error bars 95% CI.
